# Retinoic Acid Signaling Plays a Crucial Role in Excessive Caffeine Intake-Disturbed Apoptosis and Differentiation of Myogenic Progenitors

**DOI:** 10.3389/fcell.2021.586767

**Published:** 2021-03-09

**Authors:** Nian Wu, Yingshi Li, Xiangyue He, Jiayi Lin, Denglu Long, Xin Cheng, Beate Brand-Saberi, Guang Wang, Xuesong Yang

**Affiliations:** ^1^Division of Histology and Embryology, International Joint Laboratory for Embryonic Development and Prenatal Medicine, Medical College, Jinan University, Guangzhou, China; ^2^Key Laboratory for Regenerative Medicine of the Ministry of Education, Jinan University, Guangzhou, China; ^3^Department of Pathology, Medical School, Jinan University, Guangzhou, China; ^4^Department of Anatomy and Molecular Embryology, Institute of Anatomy, Ruhr-University Bochum, Bochum, Germany

**Keywords:** caffeine, myogenesis, somitogenesis, apoptosis, chicken emrbyo, retinoic acid signaling

## Abstract

Whether or not the process of somitogenesis and myogenesis is affected by excessive caffeine intake still remains ambiguous. In this study, we first showed that caffeine treatment results in chest wall deformities and simultaneously reduced mRNA expressions of genes involved in myogenesis in the developing chicken embryos. We then used embryo cultures to assess in further detail how caffeine exposure affects the earliest steps of myogenesis, and we demonstrated that the caffeine treatment suppressed somitogenesis of chicken embryos by interfering with the expressions of crucial genes modulating apoptosis, proliferation, and differentiation of myogenic progenitors in differentiating somites. These phenotypes were abrogated by a retinoic acid (RA) antagonist in embryo cultures, even at low caffeine doses in C2C12 cells, implying that excess RA levels are responsible for these phenotypes in cells and possibly *in vivo*. These findings highlight that excessive caffeine exposure is negatively involved in regulating the development of myogenic progenitors through interfering with RA signaling. The RA somitogenesis/myogenesis pathway might be directly impacted by caffeine signaling rather than reflecting an indirect effect of the toxicity of excess caffeine dosage.

## Introduction

Caffeine intake during pregnancy has been reported to be potentially teratogenic. On the one hand, both the metabolic rate and the half-life of caffeine significantly increase in maternity (Yu et al., 2016), but on the other hand, the lipophilic characteristic of caffeine allows it to transfer freely across all biological membranes, including the placental barrier (Grosso and Bracken, 2005). Therefore, maternal caffeine intake during pregnancy increases the risk of poor birth outcomes. For example, current studies in humans have shown that the birth defects induced by maternal caffeine intake include low birth weight, childhood overweight, and cognitive impairment (Qian et al., 2019). The caffeine exposure has been also reported to disrupt chondrogenesis and osteogenesis (Wink et al., 1996; Reis et al., 2013). As far as we know, muscle and cartilage cells are derived from the somites during the early stage of embryonic development (Christ and Ordahl, 1995; Jang and Baik, 2013). However, it still remains elusive whether or not the differentiation of somitic precursor populations of muscle cells as well as the myogenesis can be affected by caffeine exposure.

Mouse, rat, and chicken embryos have been extensively used as the models for studying congenital birth defects in different organ systems that have been induced by teratogen exposure (Dangata and Kaufman, 1997; Bupp Becker and Shibley, 1998; Illanes et al., 1999; Cavieres and Smith, 2000; Debelak and Smith, 2000; Roda-Moreno et al., 2000; Miller et al., 2001; Sawada et al., 2002). The chicken embryo is actually a leading model in somitogenesis studies and many of the landmark experiments, which greatly contribute to our current understanding of the process of vertebrate segmentation (Pourquié, 2004). During the early stages of chicken embryo development, somites develop from the paraxial mesoderm and constitute the segmental pattern of the body (Christ and Ordahl, 1995). They are formed in pairs by epithelialization and located on both sides of the neural tube. Somite formation results from a tight spatiotemporal control of expressions of “oscillating genes,” including the Notch signaling pathway, Wnt/beta-catenin signaling pathway, fibroblast growth factors (FGFs), Hox genes, and retinoic acid (RA) (Borycki et al., 2000; Dubrulle et al., 2001; Carapuco et al., 2005; Hamade et al., 2006). The subsequent compartmentalization of the somites is accomplished by signals emerging from neighboring tissues (Brand-Saberi et al., 1996). Diffusible factors positively or negatively control apoptosis during development. Wnts, Bone morphogenetic proteins (BMPs), and N-cadherin are implicated in dermomyotome differentiation (Capdevila et al., 1998; Linask et al., 1998), while Sonic hedgehog (Shh) is the main notochord-derived factor responsible for sclerotome differentiation (Münsterberg et al., 1995). Shh positively controls the survival of mesoderm-derived somite cells and is also capable of inducing myotomal markers in combination with the Wnts (Münsterberg et al., 1995; Marcelle et al., 1997; Cotrina et al., 2000). Apart from requirement for somite differentiation, inhibition of BMP4 signaling was reported to be important in myotomal development as well (Reshef et al., 1998). Noggin, a BMP4 antagonist, can be activated by Shh and Wnt signaling pathways (Hirsinger et al., 1997).

The earliest muscle-specific markers expressed in the somites are members of the myogenic regulatory gene family (MRF) encoding basic helix–loop–helix transcription factors, which comprise MyoD (Myod1), Myf5, MRF4, and myogenin. Myoblasts are committed to the skeletal muscle lineage by MyoD and Myf5 expression, while MRF4 and myogenin are involved in the initiation of differentiation of myogenic progenitors into myoblasts (Bober et al., 1994; Jang and Baik, 2013). The Pax gene family plays key roles in the formation of living tissues and organs during embryogenesis, of which Pax3 and Pax7 mark myogenic progenitor cells (Relaix et al., 2005; Buckingham and Relaix, 2007). Pax7 is also uniquely indispensable for satellite cells, which arise from a population of muscle progenitor cells that originate in the central domain of the dermomyotome (McKinnell et al., 2008). Myf5 has been reported to be directly regulated by Pax7 in the differentiation of satellite cell-derived myoblasts (McKinnell et al., 2008).

Using chicken embryos, our previous studies have revealed the effects of caffeine exposure on the development of the fetal nervous system, eye, and angiogenesis (Ma et al., 2012, 2014, 2016; Wang et al., 2019). Since there were no reports about caffeine influence on the process of myogenesis and somitogenesis, we set out to explore the effects and underlying biological mechanisms of caffeine on the critical time period of myogenesis during the early embryo development using the chicken embryo as a model system.

## Materials and Methods

### Experimental Chicken Embryos

Fertilized Leghorn eggs were obtained from the Avian Farm of the South China Agriculture University (Guangzhou, China) and incubated until reaching the required developmental stage (Hambuger and Hamilton) in a humidified incubator (Hamburger and Hamilton, 1951). For the caffeine-treated early stage embryos, the HH4 chicken embryos (about 12-h incubation) in early chick (EC) culture were treated with 300 μM of caffeine (Nacalai, Japan), 300 μM of caffeine + 10^−5^ M of AGN (AGN, an active RA receptor antagonist AGN193109), or same the amount of phosphate-buffered saline (PBS) until the experimentally required developmental stage. Moreover, the antibiotics penicillin/streptomycin were added to the EC culture (Chapman et al., 2001). For the elder stage embryos, HH10 (about 1.5-day incubation) pre-incubated chicken embryos were exposed to 5, 10, 15, 30 μmol/egg caffeine, or same the amount of avian saline through injection into windowed eggs *in vivo* (Ma et al., 2012, 2014, 2016; Wang et al., 2019). The surviving embryos were harvested for further analyses.

### Explant Culture

The somites and presomitic mesoderm (PSM) absence of the neural tube were separated from HH10 chicken embryos as previously reported (Wang et al., 2015). The explant were incubated in DMEM–F12 culture medium (Gibco, Grand Island, NY) or 300 μM of caffeine DMEM–F12 inside an incubator (Galaxy S; RS Biotech, Scotland, UK) at 37°C and 5% CO2 for 24 h.

### Cell Culture and Scratch Wound Migration Assay

C2C12 cells were purchased from Guangzhou Jennio Biotech Co., Ltd, China, and cultured in culture medium (DMEM–F12 GIBCO). A “scratch wound” was created by scraping a monolayer culture of C2C12 cells using a sterile 10-μl pipette tip; the images were taken using an inverted microscope (Nikon Eclipse TiU, Japan). The length of the wound gap was measured using Image pro-Plus software. The assays were performed three times using triplicate culture well.

### Histology

E6 (HH28) and E9 (HH35) chicken embryos were fixed in 4% paraformaldehyde at 4°C for 24 h. The specimens were then dehydrated, cleared in xylene, and embedded in paraffin wax before being serially sectioned at 5 μm using a rotary microtome (Leica RM2126RT, Germany). The sections were stained with hematoxylin and eosin (H&E) (Sigma-Aldrich, USA), Safranin O (Sigma-Aldrich, USA), and Masson (Sigma-Aldrich, USA) following standardized protocols. The frozen sections were prepared by sectioning at thickness of 15–20 μm on a cryotome (Leica CM1900, Bensheim, Germany).

### Immunofluorescence Staining

The harvested HH10 and E2.5 (HH17) chicken embryos were fixed in 4% paraformaldehyde overnight at 4°C. Whole-mount embryo immunostaining was performed using the following antibodies: c-Caspase3 (cleaved Caspase3, 1:200, Cell Signaling Technology, Danvers, MA), Pax7 (1:200, DSHB, Iowa City, IA), phospho-Histone H3 (PH3, 1:200, Cell Signaling Technology, USA), and MF-20 (1:200, DSHB, Iowa City, IA). Briefly, the fixed embryos were incubated with primary antibody overnight at 4°C on a shaker. After extensive washing, the embryos were incubated with either anti-mouse IgG conjugated to Alexa Fluor 555 or anti-rabbit IgG conjugated to Alexa Fluor 488 (1:1,000; Invitrogen, Waltham, MA, USA) overnight at 4°C on a shaker. All the embryos were later counterstained with DAPI (1:1,000; Invitrogen, Waltham, MA, USA) at room temperature for 1 h.

### *In situ* Hybridization

Whole-mount *in situ* hybridization of chicken embryos was performed according to a standard *in situ* hybridization protocol (Henrique et al., 1995). Briefly, digoxigenin-labeled probes were synthesized again BMP4 (Chapman et al., 2004), Wnt3a (Chapman et al., 2004), Shh (Chapman et al., 2004), Myf5, and RALDH2. RNA antisense Myf5 and RALDH2 probes used were obtained by reverse transcription polymerase chain reaction (RT-PCR) technique as described by Bales et al. (1993). Specific primers are described in Supplementary Table 1. Total RNAs were isolated from HH10 chicken embryos.

### RNA Isolation and Quantitative PCR

Total RNA was extracted using a TRIzol kit (Invitrogen, Waltham, MA, USA), from the whole HH10 chicken embryos, somites and PSM explants, C2C12 cells, or the E6 and E9 embryos after removing the head, viscera, and cartilage. First-strand cDNA was synthesized to a final volume of 20 μl using a SuperScript RIII first-strand kit (Invitrogen, USA). Following RT, PCR amplification of the cDNA was performed using chicken specific primers. Specific primers are described in Supplementary Table 1. The PCRs were performed in a Bio-Rad S1000™ Thermal cycler (Bio-Rad, Hercules, CA, USA) as the manufacturer described. The final reaction volume of 50 μl is composed of 1 μl of first-strand cDNA, 25 μM of forward primer, 25 μM of reverse primer, 10 μl of PrimeSTAR™ Buffer (Mg^2+^ plus), 4 μl of dNTPs Mixture (Takara, Tokyo, Japan), and 0.5 μl of PrimeSTAR™ HS DNA Polymerase (2.5 U/μl; Takara, Japan) in RNase-free water. The cDNAs were amplified for 30 cycles. One round of amplification was performed at 94°C for 30 s, at 58°C for 30 s, and at 72°C for 30 s. The PCR products (20 μl) were resolved in 1% agarose gels (Biowest, Hong Kong, China) in 1× TAE buffer (0.04 M of triacetate and 0.001 M of EDTA), and 10,000× GeneGreen Nucleic Acid Dye (Tiangen, Beijing, China) solution. The resolved products were visualized in a transilluminator (Syngene, Cambridge, UK), and photographs were captured using a computer-assisted gel documentation system (Syngene). The expression of genes was normalized to GAPDH. At least 15 HH10 embryos or explants were mixed for one sample; at least three E6 or E9 embryos were mixed for one sample. The quantitative PCR results shown are representative of three independent experiments.

### Western Blot

Chest wall tissues (removed cartilage) were collected from E9 control of 300 μM caffeine-treated chicken embryos. Western blot analysis was performed in accordance with the standard procedure using the c-Caspase3 (cleaved Caspase3, 1:500, Cell Signaling Technology, Danvers, MA). The protein was isolated using a radioimmunoprecipitation assay (Sigma-Aldrich) buffer supplemented with protease inhibitor. Protein concentrations were quantified with the bicinchoninic acid assay. The loading control was a β-actin antibody (1:3,000; Proteintech, Rosemont, IL). Quantity One (Bio-Rad) was used to capture the chemiluminescent signals and analyze the data. All samples were performed in triplicate.

### Enzyme-Linked Immunosorbent Assay

ELISA kits (Meibiao Biol Tech, Jiangsu, China) were used to measure RA from HH10 chicken embryos according to the manufacturer's instructions. The results were calculated using interpolation from a standard curve created by a series of RA concentrations.

### Protein–Protein Interaction Network Analysis

STRING (Search Tool for the Retrieval of Interacting Genes/Proteins, https://string-db.org) was used for the protein–protein interaction (PPI) network analysis.

### Photography

Following immunofluorescence staining or *in situ* hybridization, whole-mount embryos were photographed using a stereo-fluorescent microscope (Olympus MVX10) and associated Olympus software package Image-Pro Plus 7.0. The embryos were sectioned into 14-μm-thick slices using a cryostat microtome (Leica CM1900), and sections were photographed using an epifluorescence microscope (Olympus LX51, Leica DM 4000B) with the CN4000 FISH Olympus software package.

### Data Analysis

Data analyses and construction of statistical charts were performed using the GraphPad software (La Jolla, CA, USA). All data were expressed as the mean value (mean ± SEM). Statistical evaluation was performed using ANOVA with Tukey's pairwise comparisons or independent samples *t*-test. Statistical significance was defined as *P* < 0.05. The data are indicated with ^*^ for *P* < 0.05, ^**^ for *P* < 0.01, and ^***^ for *P* < 0.001. All the details on statistics are summarized in the Supplementary Material.

## Results

### Excess Caffeine Exposure Causes Chest Wall Deformities in Chicken Embryos

In our previous study on the effects of caffeine on the monoamine neurotransmitter system, we have investigated the effect of low doses of caffeine (i.e., 2.5, 5, and 10 μmol/egg) on the development of chicken embryos (Li et al., 2012). To obtain the adequate numbers of alive embryos with significant phenotype, we exposed HH10 (E1.5) chicken embryos with 5, 10, 15, and 30 μmol/egg caffeine every 48 h, and the embryos were harvested on the 9th day (Supplementary Figure 1A). The results manifested that caffeine exposure increased embryo mortality in a dose-dependent manner (Supplementary Figure 1B). The death rate of embryos increased to around 50% when the caffeine concentration reached 15 μmol/egg. The harvested chicken embryos were treated with 15 μmol/egg caffeine and PBS (control) for further investigations.

We first checked the weight of the 9-day-old embryos. The embryo weight of chicken embryos in the caffeine-treated group was about 20% lower than that in the control group (Supplementary Figure 1C). Actually, the embryo weight decreased from Day 6 (Supplementary Figure 3A). The morphological phenotypes of the embryos harvested on Day 9 were examined; and more than 75% of the embryos in the caffeine-treated group showed varying degrees of chest wall deformities (Supplementary Figures 1D,E). H&E, Safranin O, and Masson staining were performed on the transverse serial sections of both control and caffeine-treated embryos with mild chest wall deformities. Cartilage territorial matrix (keratan sulfate and chondroitin sulfate) is much basophilic on H&E staining. Safranin O is a basic dye that stains cartilage (proteoglycans, chondrocytes, and type II collagen) in varying shades of red. Cartilage and collagenous fiber are colored in blue, while muscle tissue is colored in red on Masson staining. The results demonstrated a significant suppression of cartilage fusion (red arrows in Supplementary Figures 2J1,N,P) and muscle tissue anomaly (Masson staining, blue arrow in Supplementary Figure 2P), indicating that chest wall development is affected by directly exposing the developing chicken embryos to high doses of caffeine.

Among the tissues affected by the deformity, muscles were particularly reduced in size. We therefore chose to examine the expression of the key genes involved in the myogenesis. Quantitative PCR data indicated that the mRNA expressions of MYH7B (a myosin heavy chain essential for the thick filaments of striated muscle), MEF2A [a myocyte enhancer factor (MEF) significant in muscle differentiation], myogenin (controlling the terminal differentiation of myoblasts into myocytes), Myod1 (muscle regulatory factor), and Pax7 (marking myogenic progenitor cells and regulating their entry into the program of skeletal muscle differentiation) were reduced in the presence of caffeine (Supplementary Figure 2Q). We further examined the expression of these genes in E6 chicken embryos, and we found a similar decreasing trend (Supplementary Figure 3B). These results are consistent with the reduction in muscle size observed by histology.

### Caffeine Treatment Suppressed the Somitogenesis of Chicken Embryos During Embryogenesis

**Figure 1:**
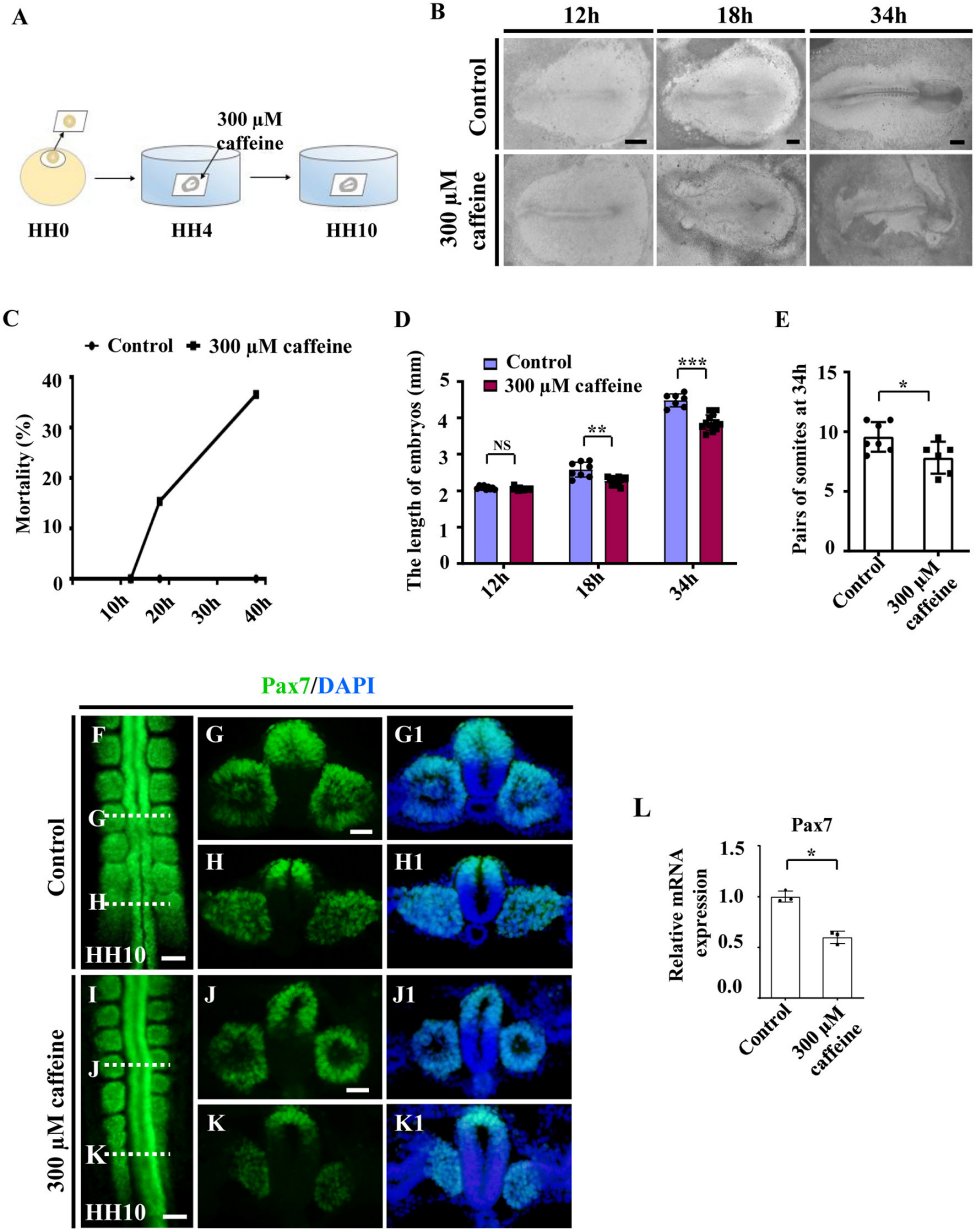
Assessment of chicken somitogenesis following caffeine treatment. **(A)** The sketch illustrates the development of chicken embryos in early chick (EC) culture with or without caffeine. Caffeine was applied to the embryos at HH4 (12-h incubation). **(B)** Representative images of whole chicken embryos from the control and caffeine-treated groups, taken after 12, 18, and 34 h of incubation. **(C)** The curve chart showing the comparisons of mortality of chicken embryos from the control and caffeine-treated groups. **(D,E)** The bar charts showing the comparisons of length and somite pairs of chicken embryos between the control and caffeine-treated groups. **(F–K, G1–K1)** Immunofluorescence staining of Pax7 in the chicken embryos from the control **(F)** and caffeine-treated **(I)** groups, and the corresponding transverse sections for Pax7 and merge with DAPI staining **(G,H,J,K,G1,H1,J1,K1)**. Panels **(G/J)** and **(H/K)** are from the seventh somites and presomitic mesoderm (PSM) of somites, respectively. **(L)** Quantitative RT-PCR data showing the mRNA expression of Pax7 from the control and caffeine-treated groups. Scale bars = 500 μm in panel **(B)**; 100 μm in panels **(F**, **I)**; 50 μm in panels **(G,H,J,K,G1,H1,J1,K1)**. *P < 0.05, **P < 0.01, ***P < 0.001.

Since the majority of the musculoskeletal system derives originally from the somites, we then investigated the effect of caffeine on somitogenesis. The HH4 chicken embryos (normally pre-incubated for about 12 h) were incubated in EC culture with or without 300 μM of caffeine (300 μM *in vitro* corresponds to 15 μmol/egg as one egg is equal to 50 g) and then harvested until stage HH10 (Figure 1A). The embryo mortality (up to around 40%) was increased with time in response to caffeine exposure (Figures 1B,C). The length of the caffeine-treated embryos was generally shorter compared with the corresponding control group after 18- and 34-h incubation (Figures 1B,D). Most of the control embryos reached HH10 (10 pairs of somite stage) after 34 h of incubation, whereas the caffeine-treated embryos did not. Moreover, at 34 h, caffeine-treated embryos exhibited a reduced number of somite pairs as compared with the control group (Figure 1E).

Embryos of the caffeine-treated group were further incubated until stage HH10 and then compared with the HH10 embryos in the control group. Immunofluorescence staining of Pax7 indicated the abnormal somite formation induced by caffeine treatment (Figures 1F–K,G1,H1,J1,K1; *n* = 6 embryos in the control group and n = 12/18 embryos in the caffeine-treated group). The mRNA levels of Pax7 were clearly decreased in the somites in the presence of caffeine compared with controls (Figure 1L). These results indicated that caffeine treatment had an early effect by interfering with somitogenesis of chicken embryos.

### Caffeine Treatment Interfered With the Expressions of Crucial Genes Modulating Apoptosis, Proliferation, and Cell Differentiation in Somites

**Figure 2:**
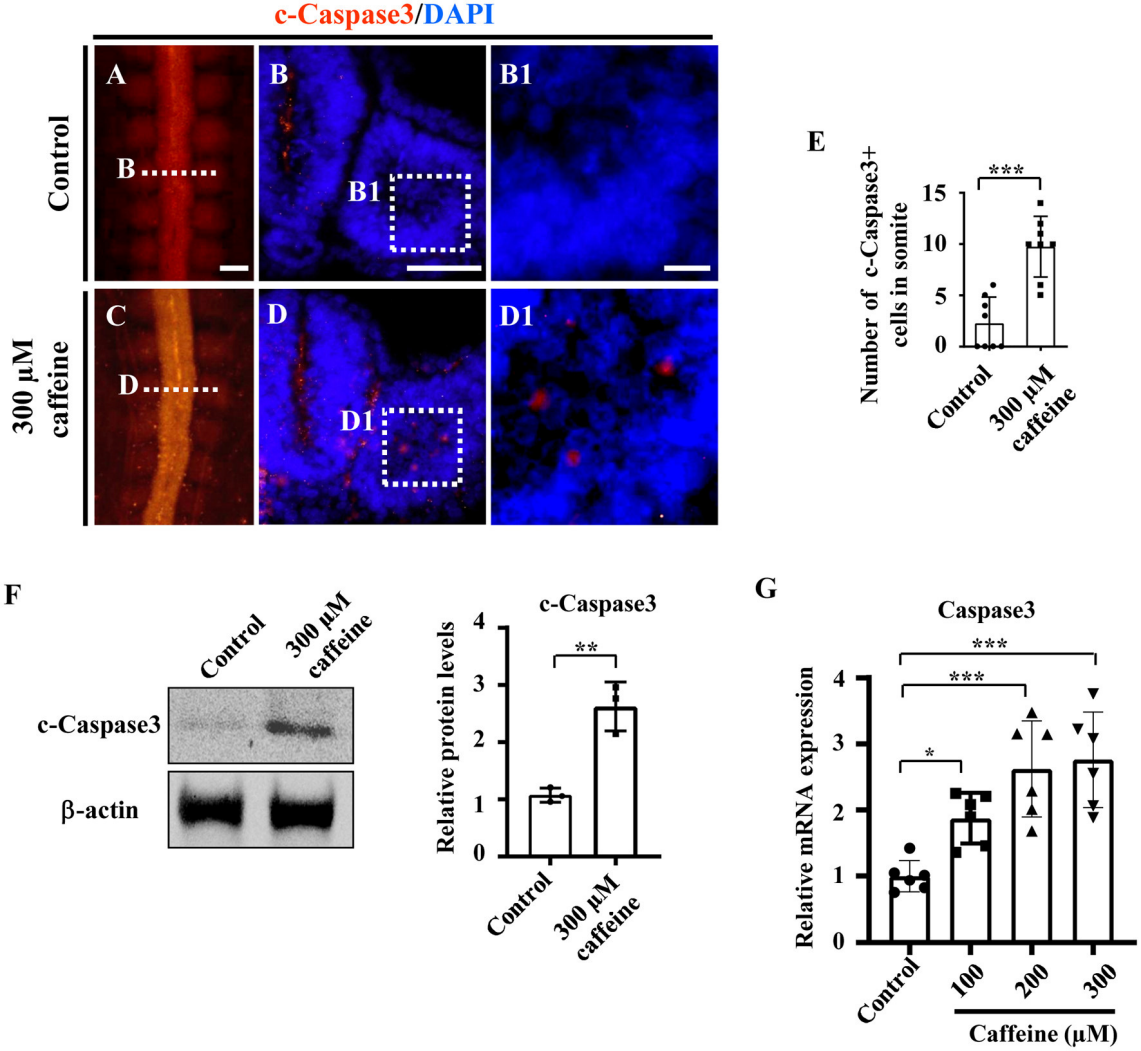
Determination of cell apoptosis in the developing somites of HH10 embryos and C2C12 cells following caffeine treatment. **(A–D)** Immunofluorescence staining of c-Caspase3 in the chicken embryos from control **(A)** and caffeine-treated **(C)** groups, and the corresponding transverse sections for c-Caspase3 and merge with DAPI staining **(B,D)**. Panels **(B/D)** is from the fifth pair of somites. **(B1,D1)** High-magnification images from the sites indicated by **(B,D)**, respectively. **(E)** The bar chart showing the proportion of c-Caspase3-positive cell numbers in somite from the control and caffeine-treated groups. c-Caspase3, cleaved Caspase3. **(F)** Western blotting showing c-Caspase3 expressions in chicken embryos without the head, viscera, and cartilage from the control and caffeine-treated groups. **(G)** Quantitative RT-PCR data showing the mRNA expression of c-Caspase3 in C2C12 cells after caffeine treatment. Scale bars = 100 μm in panels **(A,C)**; 50 μm in panels **(B,D)**; 10 μm in panels **(B1,D1)**. *P < 0.05, **P < 0.01, ***P < 0.001.

To investigate whether caffeine treatment affects the apoptosis, we used immunofluorescence c-Caspase3 staining to determine the presence of apoptotic cells in the whole embryo following caffeine treatment. The apoptosis was generalized, and the numbers of c-Caspase3-positive cells were significantly increased in the somites of the caffeine-treated group compared with the control (Figures 2A–E,B1,D1). These data indicated that caffeine treatment promoted cell apoptosis in somites. To further prove that the increased apoptosis is linked to the chest wall defect, we determined the c-Caspase3 expression using western blot in the sample from the chest wall without cartilage in E9 chicken embryos. The results indicated that c-Caspase3 expression in the chest wall from caffeine-treated embryos was much higher than the one from the control (Figure 2F). The similar results were also obtained in the mouse C2C12 cells treated with 300 μM of caffeine and lower doges of 100 and 200 μM (Figure 2G). Quantitative PCR data indicated that the Caspase3 mRNA was increased in a dose-dependent manner (Figure 2G). To investigate whether or not caffeine could directly cause the cell apoptosis of somites, we determined c-Caspase3 expression in the cultured explants composed of somite and PSM *in vitro*. The quantitative PCR data indicated that the Caspase3 mRNA levels were increased following 300 μM caffeine treatment (Supplementary Figures 4A,B). These results suggest that caffeine treatment can directly lead to cell apoptosis in somite.

In order to explore what caused the reduction of tissue size, cell proliferation was also detected on the HH10 embryos and C2C12 cells, as well as somite and PSM explants. Interestingly, the number of PH3-positive cells was significantly increased in the somites of the caffeine-treated group compared with the control (note: the red arrows indicate the PH3-positive cells in the somites) (Supplementary Figures 5A–E). Quantitative PCR data demonstrated that the Cyclin D1 mRNA levels were also increased in somite and PSM explants (Supplementary Figure 4C). The increase was observed in a dose-dependent manner in C2C12 cells (Supplementary Figure 5F). These results show that the reduced size does not result from a reduced proliferation rate, as it is unexpectedly associated with an increase in proliferation in somites.

**Figure 3:**
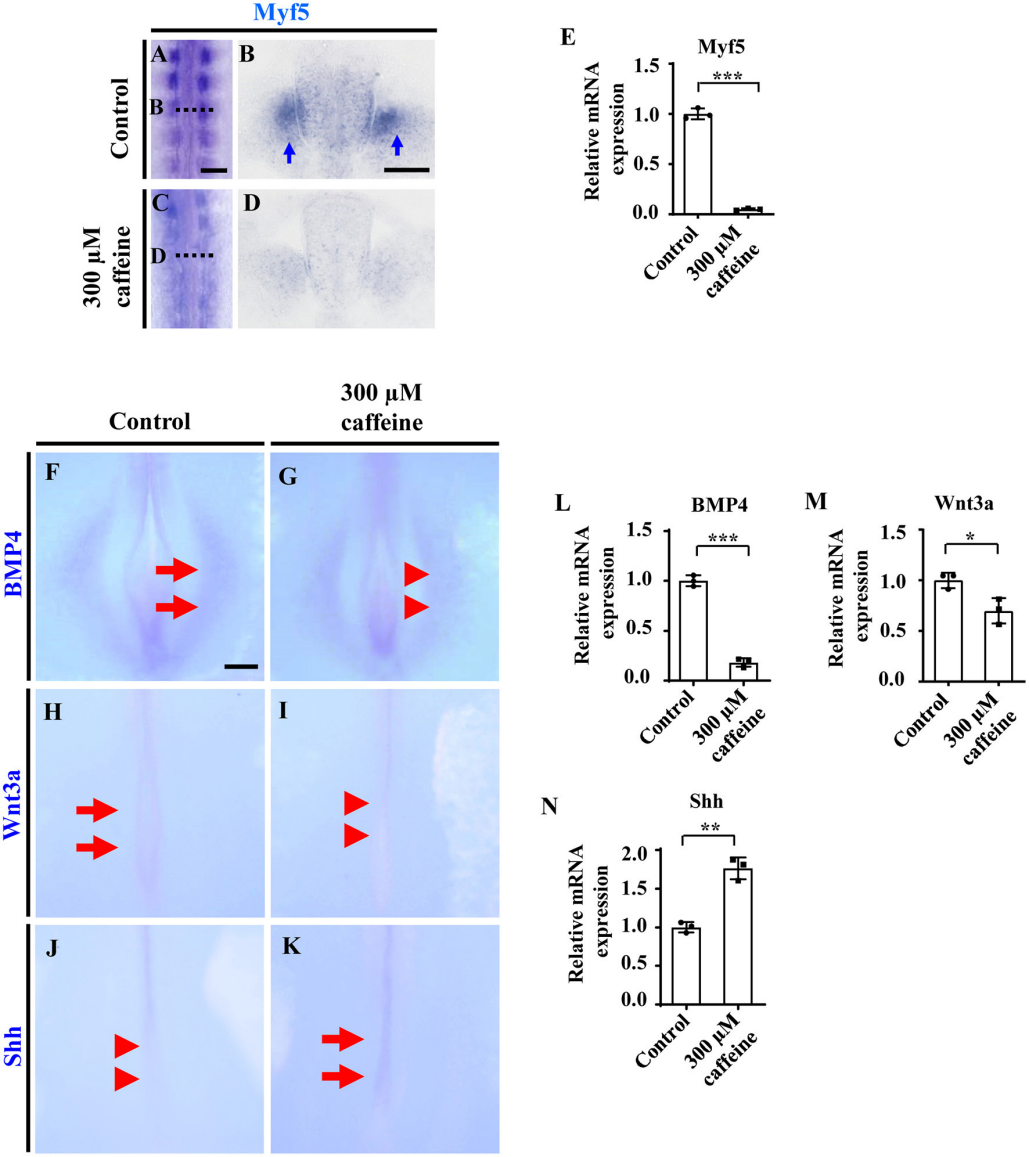
Determination of cell differentiation in the developing somites following caffeine treatment. **(A–D)** Whole-mount *in situ* hybridization for Myf5 in the chicken embryos from control **(A)** and caffeine-treated **(C)** groups, and the corresponding transverse sections **(B,D)**. Panels **(B/D)** is from the fifth pair of somites. **(E)** Quantitative RT-PCR data showing the mRNA expression of Myf5 from the control and caffeine-treated groups. **(F–K)** Whole-mount *in situ* hybridization for BMP4, Wnt3a, and Shh in the chicken embryos from control **(F,H,J)** and caffeine-treated **(G,I,K)** groups. **(L–N)** Quantitative RT-PCR data showing the mRNA expression of BMP4, Wnt3a, and Shh from the control and caffeine-treated groups. Scale bars = 100 μm in panels **(A,C)**; 50 μm in panels **(B,D)**; 200 μm in panels **(F–K)**. *P < 0.05, **P < 0.01, ***P < 0.001.

We then determined the expression of Myf5 in the somite, which is a myogenic precursor marker. The whole-mount *in situ* hybridization (Figures 3A,C), and the corresponding sections (Figures 3B,D) showed that caffeine treatment dramatically inhibited the expression of Myf5 during somite differentiation of chicken embryos. Quantitative PCR data indicated that the mRNA expression of Myf5 was also reduced in the presence of caffeine (Figure 3E and Supplementary Figures 4C, 5D).

*In situ* hybridization and quantitative PCR were carried out to determine the expression change of diffusible factors that are involved in regulating cell survival positively or negatively during development. Whole-mount *in situ* hybridization of BMP4, Wnt3a, and Shh showed that caffeine treatment significantly inhibited the expression of BMP4 (*n* = 6 embryos in the control group and *n* = 5/6 embryos in the caffeine-treated group) and Wnt3a (*n* = 6 embryos in the control group and *n* = 5/6 embryos in the caffeine-treated group) but increased the expression of Shh (*n* = 6 embryos in the control group and *n* = 4/6 embryos in the caffeine-treated group) at the posterior level in HH10 chicken embryos (red arrowheads in Figures 3G,I,J indicated the lower expression compared with the arrows in Figures 3F,H,K, indicating expressions). Quantitative PCR results verified the alteration on these gene (Figures 3L–N).

### Caffeine Treatment Leads to Dramatic Alteration of Retinoic Acid Signaling During Somite Differentiation

The asymmetries of somite pairs in caffeine-treated chicken embryos (Figures 1J,K) are reminiscent of the published research findings, which demonstrated that RALDH2 was required to ensure the symmetric production of somite pairs during somitogenesis (Kawakami et al., 2005; Vermot et al., 2005; Sirbu and Duester, 2006). Moreover, RALDH2 could influence somitogenesis and myogenesis by means of acting on Shh and BMP4 (Power et al., 1999; Mic and Duester, 2003; Pourquié, 2018). Therefore, it is reasonable to infer that caffeine treatment-suppressed somite development was achieved by interfering with RA pathway.

**Figure 4:**
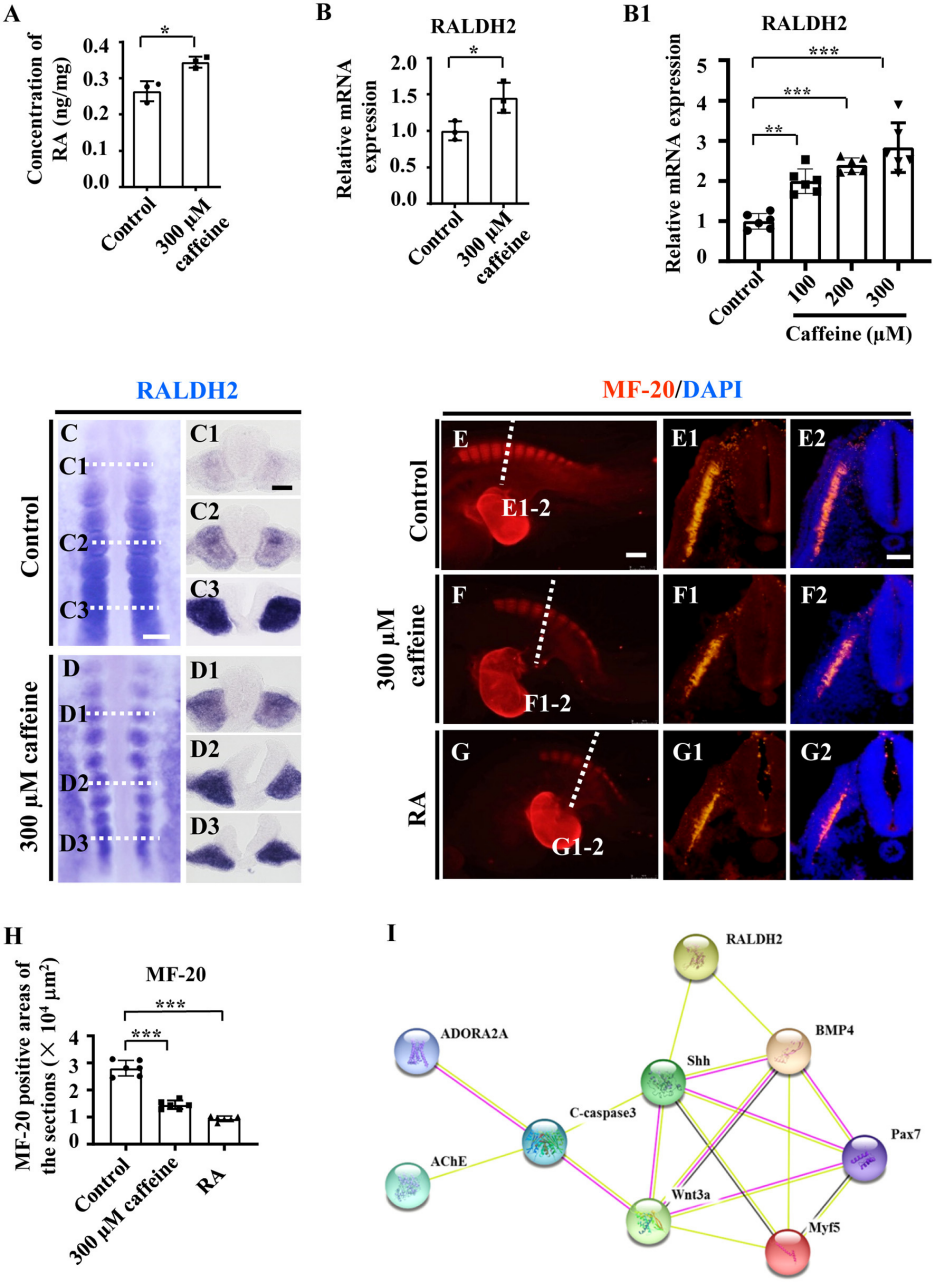
Assessment of the somitogenesis-related gene expressions following caffeine treatment. **(A)** ELISA data showing the concentration of retinoic acid (RA) in the control and caffeine-treated groups of HH10 chicken embryos. **(B,B1)** Quantitative RT-PCR data showing the mRNA expression of RALDH2 from the control and caffeine-treated groups of HH10 chicken embryos **(B)** and C2C12 cells **(B1)**. **(C,D,C1–C3,D1–D3)** Whole-mount *in situ* hybridization for RALDH2 in the chicken embryos from the control **(C)** and caffeine-treated **(D)** groups, and the corresponding transverse sections. Panels **(C1/D1,C2/D2,C3/D3)** are from the third, sixth, and ninth pairs of somites, respectively. **(E–G,E1–G1,E2–G2)** Immunofluorescence staining of MF-20 in the E2.5 chicken embryos from the control **(E)**, caffeine-treated **(F)**, and RA-treated **(G)** groups, and the corresponding transverse sections (fifth pair of somites) for MF-20 and merge with DAPI staining **(E1–G1,E2–G2)**. **(H)** Quantitative data showing the areas of MF-20 immunofluorescence staining in E1, F1, and G1. **(I)** The protein–protein interactions from the STRING database showing the network of ADORA2A, AChE, c-Caspase3, RALDH2, Shh, BMP4, Pax7, Wnt3a, and Myf5. Scale bars = 100 μm in panels **(C,D)**; 50 μm in panels **(C1–C3,D1–D3)**; 200 μm in panels **(E–G)**; 50 μm in panels **(E1–G1,E2–G2)**. *P < 0.05, **P < 0.01, ***P < 0.001.

Consistently with the link highlighted above (somite/RALDH2), these abnormalities in somite development are associated with an increase in RALDH2 levels. These findings are further corroborated by the observation by ELISA. The ELISA data showed that the RA concentration (RA synthesis being ensured by RALDH2) was increased in caffeine-treated HH10 chicken embryos (Figure 4A). Accordingly, the mRNA levels of RALDH2 in chicken embryos, C2C12 cells, as well as somite and PSM explants, were significantly enhanced (Figures 4B,B1 and Supplementary Figures 4D, 5F). The caffeine-induced increase of RALDH2 expression in chicken embryos was verified by RALDH2 whole-mount *in situ* hybridization and corresponding transverse sections (Figures 4C,D,C1–D3). RALDH2 expression was significantly increased on somites at C1/D1 and C2/D2 levels in the caffeine-treated group compared with the control group (*n* = 5 embryos in the control group and *n* = 5/7 embryos in the caffeine-treated group). However, the RALDH2 expression at C3/D3 level seemed to get weaker in some of the embryos in the caffeine-treated group (*n* = 5 embryos in the control group and *n* = 3/7 embryos in the caffeine-treated group). These results revealed that the total mRNA level of RALDH2 in chicken embryos was promoted in the presence of caffeine, especially obvious after the somite formation.

Moreover, we used immunohistochemistry to determine the expression levels of the myosin marker MF-20 in the control, caffeine-treated, and RA-treated E2.5 chicken embryos. The results showed that MF-20 expression was significantly decreased in both groups compared with the control (Figures 4E–H,E1–G1,E2–G2). All the data indicate that RA signaling could be involved in caffeine-induced inhibition of somitogenesis and subsequent compartment formation/differentiation of somite during the early chicken embryo development. These matched the finding from the bioinformatics approach through exploring the relationships in the context of pharmacological target proteins of caffeine [adenosine A2A receptor gene (ADORA2A) and acetylcholinesterase (AChE)], apoptosis, and somitogenesis/myogenesis proteins (Figure 4I).

**Figure 5:**
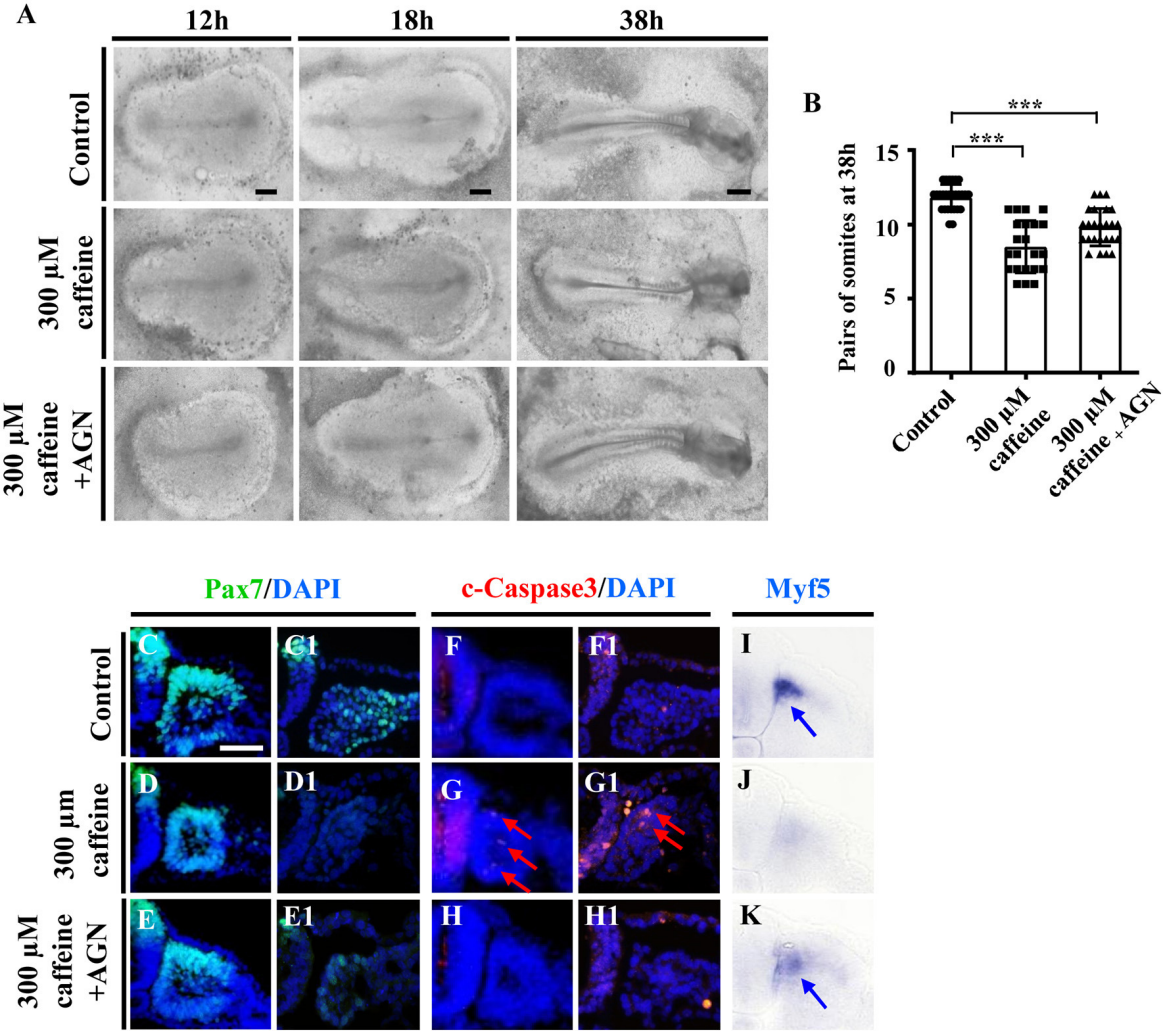
Determination of the expression of cell apoptosis and differentiation in the developing somites following caffeine treatment and blockage of retinoic acid (RA) signaling. **(A)** Representative images of the 12th-, 18th-, and 38th-h whole chicken embryos from the control, caffeine-treated, and caffeine+AGN-treated groups. **(B)** The bar charts showing the comparisons of somite pairs chicken embryo between the control, caffeine-treated, and caffeine+AGN-treated groups at 38 h. **(C–H)** Immunofluorescence staining of Pax7 or c-Caspase3 in the transverse sections from the control **(C,C1,F,F1)**, caffeine-treated **(D,D1,G,G1)**, and caffeine+AGN-treated **(E,E1,H,H1)** groups, and merge with DAPI staining. **(I–K)** The transverse sections of whole-mount *in situ* hybridization for Myf5 in the chicken embryos from the control **(I)**, caffeine-treated **(J)**, and caffeine+AGN-treated **(K)** groups. Panels **(C/D/E,F/G/H,I/J/K)** are from the seventh pairs of somites; (**C1/D1/E1,F1/G1/H1)** are from the presomitic mesoderm (PSM) of somites. Scale bars = 500 μm in panel **(A)**; 50 μm in panels **(C–K)**. ***P < 0.001.

To further confirm that the RA signaling plays an important role in caffeine disturbed somitogenesis and myogenesis, we detected whether or not the caffeine-induced abnormalities of somite development could be rescued by AGN (a potent RA receptor antagonist) (Bayha et al., 2009). We exposed the chicken embryos to caffeine and/or the AGN, and we found that blocking the RA signaling with AGN could significantly rescue the caffeine-induced reductions of embryo length and somite pairs (Figures 5A,B). The data from immunofluorescence staining and *in situ* hybridization manifested that the reduction of Pax7 and Myf5 expression caused by caffeine could be reversed by adding AGN (Pax7: *n* = 5 embryos in the control group, *n* = 4/5 embryos in the caffeine-treated group, and *n* = 5/6 embryos in the caffeine+AGN group; Myf5: *n* = 5 embryos in the control group, *n* = 5/5 embryos in the caffeine-treated group, and *n* = 6/6 embryos in the caffeine+AGN group), whereas the increased expression of c-Caspase3 caused by caffeine could be reversed by adding AGN (*n* = 5 embryos in the control group, *n* = 5/5 embryos in the caffeine-treated group, and *n* = 6/6 embryos in the caffeine+AGN group) (Figures 5C–H,C1–H1,I–K). Similar results were obtained on C2C12 cells after adding AGN (Supplementary Figures 4A–C,E). Taken together, these results indicated that RA signaling played an indispensable role in the negative effects of caffeine on somitogenesis and myogenesis (Grant et al., 2018).

**Figure 6:**
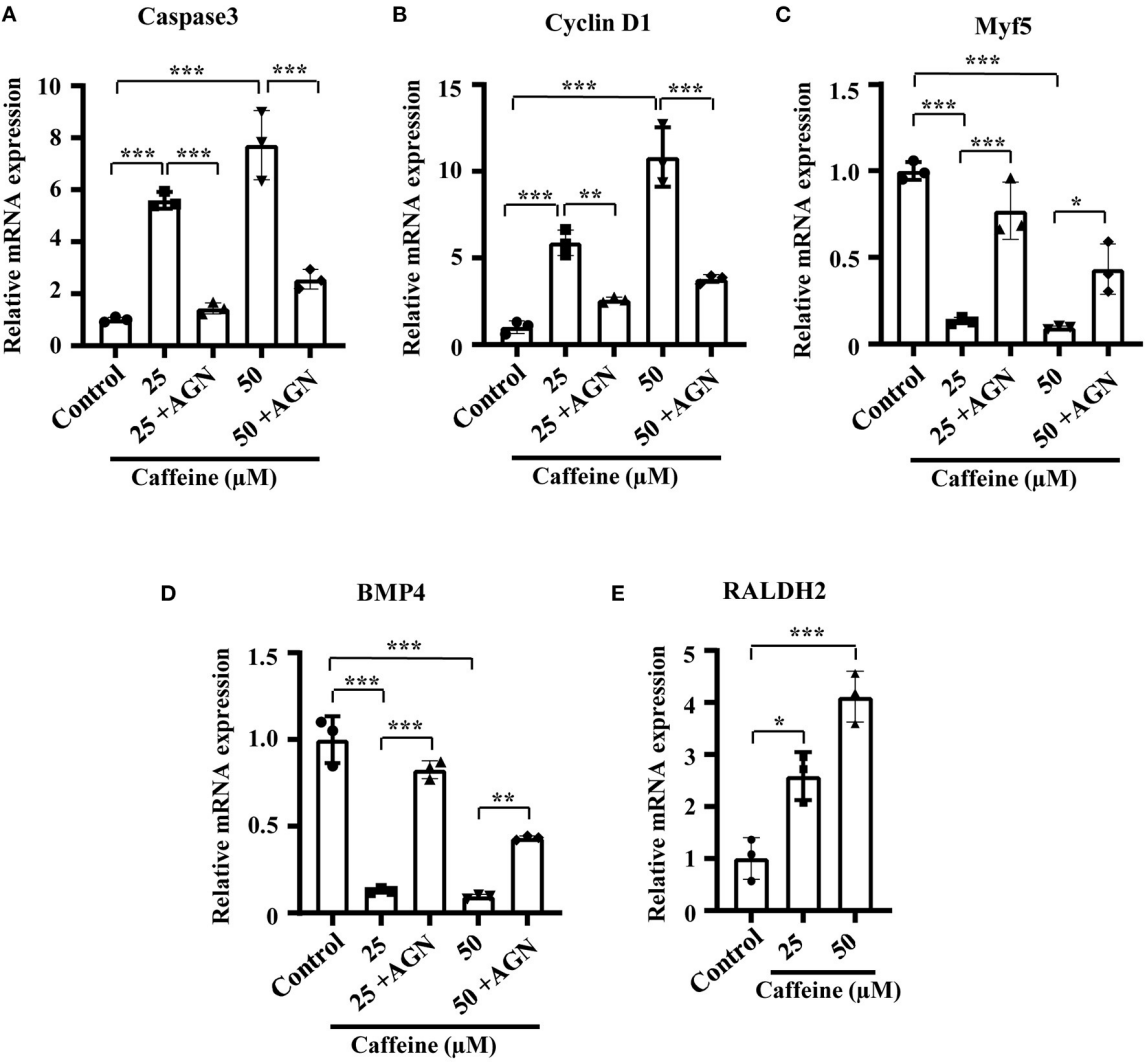
Quantitative RT-PCR data of Caspase3 **(A)**, Cyclin D1 **(B)**, Myf5 **(C)**, RALDH2 **(D)**, and BMP4 **(E)** in C2C12 cells after caffeine treatment and blockage of retinoic acid (RA) signaling. *P < 0.05, **P < 0.01, ***P < 0.001.

In order to investigate whether the phenotypes and the mechanism involving RA signaling can be uncoupled from the lethality caused by toxic levels of caffeine, we determined the effect of caffeine at low doses on C2C12 cells after adding AGN. At 25 and 50 μM (which are equal to 1.25 and 2.5 μmol/egg, respectively), the increased RNA levels of Caspase3 and Cyclin D1 as well as the decreased levels of Myf5 and BMP4 can be reverted with the RA antagonist AGN (Figures 6A–D). The RALDH2 RNA levels were also increased after lower dosages of caffeine treatment (Figure 6E). These results suggest that the elevated RA levels have a probability of occurring as a cellular signaling response to caffeine exposure. These observed regulatory changes may not be directly caused by the caffeine toxicity.

## Discussion

Caffeine is a widely consumed psychostimulant all around the world, and its intake by adults mainly comes from coffee, cola drinks, and tea (Nehlig, 2018). Growing data have shown that the daily consumed product implies negative affect on both central nervous system and cardiovascular system (Nehlig et al., 1992; Zulli et al., 2016; Grant et al., 2018). It has also been reported that caffeine can easily penetrate the mammalian placenta and can increase the risk of causes of teratogenesis in young fetuses (Santos and Lima, 2016; Yadegari et al., 2016). Caffeine consumption (>300 mg/day) during pregnancy was associated with an increased risk of fetal growth restriction, and this association continued throughout pregnancy. Interestingly, the assessment of the more physiologically relevant doses of caffeine on embryonic development and uterine receptivity in mice indicated that there was no effect at low doses (2 mg/g), but effects started at 5 mg/g (Qian et al., 2018). In rats, maternal coffee consumption could cause the higher accumulation of caffeine in fetal brain than in serum (Wilkinson and Pollard, 1993). Since 100–600 μM of caffeine could affect neurogenesis in the mouse model (Narod et al., 1991; Marret et al., 1997), we applied 15 μmol/egg of caffeine (each egg contains about 50 ml of albumen and yolk) or EC culture mixed with 300 μM of caffeine based on our previous study (Ma et al., 2012, 2014, 2016; Wang et al., 2019). We do not deny that the concentration of caffeine is hardly representative of the conditions of high caffeine exposure during pregnancy. But we speculate that the caffeine doses are within the acceptable range in acute toxicity studies (note: the chicken embryo develops and hatches in 20–21 days only).

In this study, by using the chicken embryo model, we found that caffeine exposure reduced the weight of developing chicken embryos and also induced a delay in somite formation and chest wall malformations (Supplementary Figures 1C–E and Figures 1D,E). In fact, the chest wall comprises fat, skin, muscles, and the thoracic skeleton (Clemens et al., 2011). Congenital chest wall deformities are considered to be anomalies in chest wall growth and can be seen with various anomalies of the musculoskeletal system and collagen fibers (Mak et al., 2016). Abnormal cartilage fusion and muscle tissue formation were very obvious in the caffeine-treated group with mild unclosed chest wall (Supplementary Figures 2J1,N,P).

Moreover, there was a significant reduction in mRNA expressions of myogenesis-related genes including MYH7B, MEF2A, Myod1, myogenin, and Pax7 (Supplementary Figure 2Q). MYH7B has been found to be expressed in a range of muscle tissues in *Xenopus*, chicken, and mouse embryos (Warkman et al., 2012). MEF2 genes (MEF2A, B, C, and D), which lack myogenic activity alone, interact with MRFs (Myf5, MRF4, Myod1, and myogenin) to synergistically activate muscle-specific genes and the myogenic differentiation program, and the two families directly regulate the expression of an extensive array of muscle structural genes (Molkentin and Olson, 1996; Blais et al., 2005). Pax7 marks myogenic progenitor cells and regulates their behavior and their the entrance program of skeletal muscle differentiation through networking with MRFs (Relaix et al., 2005; Buckingham and Relaix, 2007). All these results indicated that caffeine exposure impaired myogenesis of the early-stage embryos. It seems to correspond with the previous research on zebrafish that the caffeine exposure led to myofibril misalignment (da Costa and de Lemos Menezes, 2018).

Somites have been considered to be the precursors of the axial skeleton and skeletal muscles, and the majority of the musculoskeletal system derives originally from the somites. We then determined the effect of caffeine on somitogenesis. The HH4 embryos treated with caffeine to 18th and 34th hour (HH10) exhibited significant reduction in both length of embryos and pairs of somites (Figures 1D,E). The somite defects could also be easily observed by Pax7 immunofluorescence staining (Figures 1J,K). We had employed Pax7 to study the effects of caffeine exposure on neural crest cell migration, as Pax7 is also the one of the earliest markers of avian neural crest cells and expressed at the dorsal side of the neural tube and migratory neural crest cells (Ma et al., 2012). The diversity of Pax7 expression might be due to the different kinds of cells and/or the different stages of embryos. Moreover, we found some abnormalities caused by caffeine treatment on the regions of the head (Figure 5A). The somite and PSM explants, as well as the *in vitro* experiments of myoblast cells, suggest that caffeine could affect the somite development directly (Supplementary Figure 4). Also, the chicken embryos required longer time to reach HH10 stage (36 h) compared with the control (34 h). These results suggest that excessive caffeine exposure can cause the disturbance on somitogenesis in chicken embryonic development.

The next aim was to study the pathological mechanism of embryonic myogenesis disturbance by caffeine. First, caffeine exposure could promote cell apoptosis in the somites (Figures 2A–E,B1,D1), which might be partially responsible for caffeine-induced defects in somitogenesis and myogenesis. These might be generally similar to the finding that caffeine induces apoptosis through p53, Bax, and Caspase3 pathways (He et al., 2003). Accordingly, myogenic regulatory factor Myf5 in the developing chicken embryos in the presence of caffeine was observed to be down-regulated (Figures 3A–E).

In order to explore the underlying mechanism about the reduction of tissue size induced by caffeine treatment, we then determined the potential effect of caffeine on cell proliferation. Interestingly, the result implied that the caffeine exposure played a positive role in the cell proliferation of somites (Figure 6B and Supplementary Figures 4C, 5). This finding is similar to the previous reports that caffeine promoted the proliferation of rat neuronal cells, human lung adenocarcinoma cells, and small airway epithelial cells (Al-Wadei et al., 2006; Sahu et al., 2013). Physiological cell death in the somites has been reported to be the result of the interaction of nerve growth factor with its receptor, whereas Shh induces a decrease of nerve growth factor mRNA leading to trophic support for the survival of somite cells (Teillet et al., 1998; Borycki et al., 1999; Cotrina et al., 2000; Kruger et al., 2001). Shh works together with the RA pathway to ensure the robustness of somite formation (Resende et al., 2010). Our data indicate that caffeine treatment led to the increased expressions of Shh and RA (Figures 3N, 4A). It may explain the observation that the expressions of PH3 and Cyclin D1 at mRNA levels increased following caffeine treatment (Figure 6B and Supplementary Figures 4C, 5). Apart from Shh, members of the Wnt family are implicated in muscle development (Münsterberg et al., 1995). A previous finding has shown that the expression of BMP4 is increased with progression of myogenesis and that down-regulation of BMP signal components inhibits myotube differentiation and maturation (Umemoto et al., 2011). In this study, Wnt3a and BMP expressions were evidently suppressed following caffeine exposure (Figures 3L,M). It suggests that aberrant somitic differentiation induced by caffeine might partially derive from the abnormal expressions of these crucial genes and proteins involved in regulating somite differentiation. Conclusively, caffeine exposure could lead to increased Shh expression in the neural tube and notochord and then in turn activate the ectopic expression of Noggin, subsequently resulting in the blocking of BMP4 specification of the lateral somites. The blocking of BMP4 would negatively result in a reduction of Wnt3a expression.

We then determined the expression of RALDH2, which was identified as a major RA generating enzyme in the early embryo. Both RALDH2 and RA were increased significantly in the caffeine-treated group (Figures 4A–D,B1,C1–C3,D1–D3). Similar results were obtained on C2C12 cells and the isolated somites and PSM (Figure 6E and Supplementary Figure 4F). These findings correspond with the research that RA treatment induces extensive apoptosis and down-regulation of Wnt3a (Shum et al., 1999). BMP4 expression is proximally inhibited by RA signaling, and BMP signaling may be responsible for regulating the pattern of myogenic cell migration in Raldh2–/– forelimb buds (Mic and Duester, 2003). It may address the observation that the migration ability of C2C12 cells was inhibited following caffeine treatment in the scratch wound migration assay (Supplementary Figure 6).

Furthermore, the expression of myosin marker, MF-20, was observed to be reduced in caffeine and RA separately treated groups (Figures 4F–H,F1,F2,G1,G2). Caffeine and RA either separately or in combination impaired the skeleton development of mouse embryos (Lashein et al., 2016). All of these indicate that excessive RA may negatively mediate the somitogenesis and somite differentiation in the caffeine-treated group. To verify our hypothesis, a potent RA receptor antagonist, AGN, was added together with caffeine to the chicken embryos, which rescued the reduced embryo length and pairs of somites in the corresponding caffeine-treated group (Figures 5A,B). Moreover, the expressions of Pax7 and Myf5 increased and c-Caspase3 expression decreased (Figures 5C–K,C1–E1,F1–H1), which further confirmed the crucial role of RA signaling in the process. Similar results on mRNA levels of Caspase3, Cyclin D1, Myf5, and BMP4 were obtained on C2C12 cells following low doses of caffeine and AGN treatment (Figures 6A–D).

**Figure 7:**
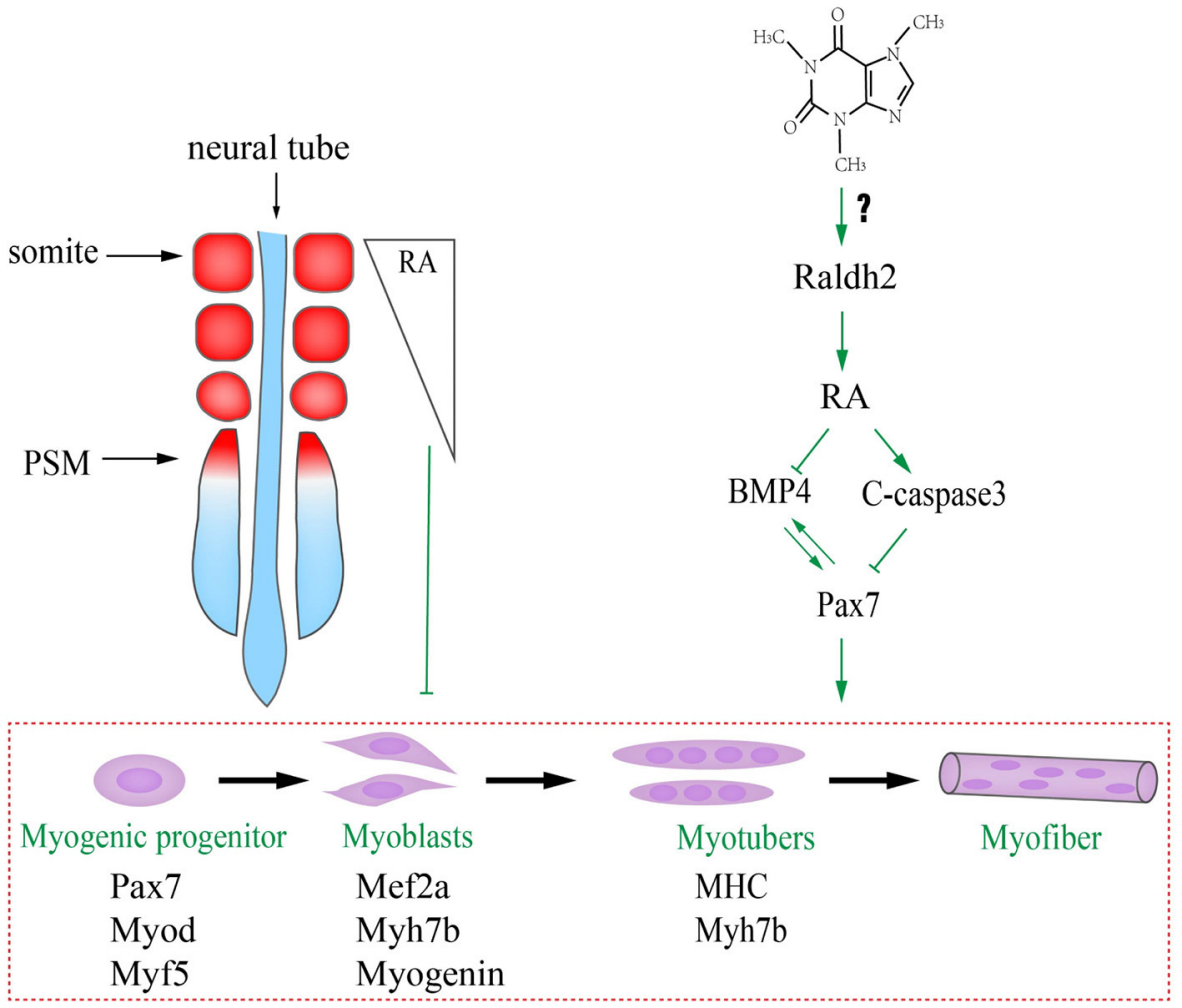
Proposed mechanism about excessive caffeine intake-disturbed differentiation of myogenic progenitors through retinoic acid (RA) signaling pathway.

We have previously reported that caffeine exposure induced its cytotoxic effects by generating excess reactive oxygen species (ROS) that could adversely affect chicken embryo development including the suppression of neurogenesis (Ma et al., 2014), and the excess ROS generation interfered with the RA production during embryogenesis (Song et al., 2020). The underlying mechanism in this study might be that caffeine disturbed the RA production during embryogenesis by increasing the RALDH2 expression (Figure 7). Consequently, the turbulence of RA metabolism, which acts as the crucial signaling to modulate the biological clock on somitogenesis and somite differentiation via influencing BMP signaling, occurred when somites form and differentiate into dermomyotome (later will convert to myotome and dermatome) and sclerotome. On the other hand, the elevated RA promotes c-Caspase3 but suppressed Pax7 expressions. Furthermore, excess caffeine exposure could lead to generalized systemic toxicity. Whether or not there is a direct link between caffeine and RA pathway needs further investigation.

## Data Availability Statement

The original contributions presented in the study are included in the article/Supplementary Material, further inquiries can be directed to the corresponding author/s.

## Ethics Statement

The animal study was reviewed and approved by Laboratory Animal Ethics Committee of Jinan University.

## Author Contributions

GW and XY designed the research project. YL, XH, JL, NW, and DL carried out the experiments and interpreted data. NW wrote the manuscript with input from GW and XY. XC, and BB-S commented on the manuscript. All authors contributed to the article and approved the submitted version.

## Conflict of Interest

The authors declare that the research was conducted in the absence of any commercial or financial relationships that could be construed as a potential conflict of interest.

## References

[B1] Al-Wadei H. A. Takahashi T. Schuller H. M. (2006). Caffeine stimulates the proliferation of human lung adenocarcinoma cells and small airway epithelial cells via activation of PKA, CREB and ERK1/2. Oncol. Rep. 15, 431–435. 10.3892/or.15.2.431 16391865

[B2] Bales K. Hannon K. Smith I. I. C Santerre R. (1993). Single-stranded RNA probes generated from PCR-derived DNA templates. Mol. Cell. Probes 7, 269–275. 10.1006/mcpr.1993.1040 8232343

[B3] Bayha E. Jørgensen M. C. Serup P. Grapin-Botton A. (2009). Retinoic acid signaling organizes endodermal organ specification along the entire antero-posterior axis. PLoS One 4:e5845. 10.1371/journal.pone.0005845 19516907 PMC2690404

[B4] Blais A. Tsikitis M. Acosta-Alvear D. Sharan R. Kluger Y. Dynlacht B. D. (2005). An initial blueprint for myogenic differentiation. Genes Dev. 19, 553–569. 10.1101/gad.1281105 15706034 PMC551576

[B5] Bober E. Brand-Saberi B. Ebensperger C. Wilting J. Balling R. Paterson B. M.. (1994). Initial steps of myogenesis in somites are independent of influence from axial structures. Development 120, 3073–3082. 7720553 10.1242/dev.120.11.3073

[B6] Borycki A. Brown A. M. Emerson C. P. Jr. (2000). Shh and Wnt signaling pathways converge to control Gli gene activation in avian somites. Development 127, 2075–2087. Available online at: https://dev.biologists.org/content/develop/127/10/2075.full.pdf 10769232 10.1242/dev.127.10.2075

[B7] Borycki A.-G. Brunk B. Tajbakhsh S. Buckingham M. Chiang C. Emerson C. (1999). Sonic hedgehog controls epaxial muscle determination through Myf5 activation. Development 126, 4053–4063. 10457014 10.1242/dev.126.18.4053

[B8] Brand-Saberi B. Wilting J. Ebensperger C. Christ B. (1996). The formation of somite compartments in the avian embryo. Int. J. Dev. Biol. 40, 411–420. 8735956

[B9] Buckingham M. Relaix F. (2007). The role of Pax genes in the development of tissues and organs: Pax3 and Pax7 regulate muscle progenitor cell functions. Annu. Rev. Cell Dev. Biol. 23, 645–673. 10.1146/annurev.cellbio.23.090506.123438 17506689

[B10] Bupp Becker S. R. Shibley I. A. Jr. (1998). Teratogenicity of ethanol in different chicken strains. Alcohol Alcohol. 33, 457–464. 10.1093/alcalc/33.5.457 9811197

[B11] Capdevila J. Tabin C. Johnson R. L. (1998). Control of dorsoventral somite patterning by Wnt-1 and β-catenin. Dev. Biol. 193, 182–194. 10.1006/dbio.1997.8806 9473323

[B12] Carapuco M. Novoa A. Bobola N. Mallo M. (2005). Hox genes specify vertebral types in the presomitic mesoderm. Genes Dev. 19, 2116–2121. 10.1101/gad.338705 16166377 PMC1221883

[B13] Cavieres M. F. Smith S. M. (2000). Genetic and developmental modulation of cardiac deficits in prenatal alcohol exposure. Alcohol. Clin. Exp. Res. 24, 102–109. 10.1111/j.1530-0277.2000.tb04559.x 10656199

[B14] Chapman S. C. Brown R. Lees L. Schoenwolf G. C. Lumsden A. (2004). Expression analysis of chick Wnt and frizzled genes and selected inhibitors in early chick patterning. Dev. Dyn. 229, 668–676. 10.1002/dvdy.10491 14991722

[B15] Chapman S. C. Collignon J. Schoenwolf G. C. Lumsden A. (2001). Improved method for chick whole-embryo culture using a filter paper carrier. Dev. Dyn. 220, 284–289. 10.1002/1097-0177(20010301)220:3<284::AID-DVDY1102>3.0.CO;2-5 11241836

[B16] Christ B. Ordahl C. P. (1995). Early stages of chick somite development. Anatomy Embryol. 191, 381–396. 10.1007/BF00304424 7625610

[B17] Clemens M. W. Evans K. K. Mardini S. Arnold P. G. (2011). Introduction to chest wall reconstruction: anatomy and physiology of the chest and indications for chest wall reconstruction. Semin. Plast. Surg. 25, 5–015. 10.1055/s-0031-1275166 22294938 PMC3140236

[B18] Cotrina M. L. González-Hoyuela M. Barbas J. A. Rodríguez-Tébar A. (2000). Programmed cell death in the developing somites is promoted by nerve growth factor via its p75NTR receptor. Dev. Biol. 228, 326–336. 10.1006/dbio.2000.9948 11112333

[B19] da Costa K. V. T. de Lemos Menezes P. (2018). Effect of caffeine on vestibular evoked myogenic potential: a systematic review with meta-analysis. Braz. J. Otorhinolaryngol. 84, 381–388. 10.1016/j.bjorl.2017.11.003 29361437 PMC9449160

[B20] Dangata Y. Y. Kaufman M. H. (1997). Morphometric analysis of the postnatal mouse optic nerve following prenatal exposure to alcohol. J. Anatomy 191 (Pt 1), 49–56. 10.1046/j.1469-7580.1997.19110049.x 9279658 PMC1467658

[B21] Debelak K. A. Smith S. M. (2000). Avian genetic background modulates the neural crest apoptosis induced by ethanol exposure. Alcohol. Clin. Exp. Res. 24, 307–314. 10.1111/j.1530-0277.2000.tb04612.x 10776667

[B22] Dubrulle J. McGrew M. J. Pourquie O. (2001). FGF signaling controls somite boundary position and regulates segmentation clock control of spatiotemporal Hox gene activation. Cell 106, 219–232. 10.1016/S0092-8674(01)00437-8 11511349

[B23] Grant S. S. Magruder K. P. Friedman B. H. (2018). Controlling for caffeine in cardiovascular research: a critical review. Int. J. Psychophysiol. 133, 193–201. 10.1016/j.ijpsycho.2018.07.001 29981767

[B24] Grosso L. M. Bracken M. B. (2005). Caffeine metabolism, genetics, and perinatal outcomes: a review of exposure assessment considerations during pregnancy. Ann. Epidemiol. 15, 460–466. 10.1016/j.annepidem.2004.12.011 15967394

[B25] Hamade A. Deries M. Begemann G. Bally-Cuif L. Genet C. Sabatier F.. (2006). Retinoic acid activates myogenesis *in vivo* through Fgf8 signalling. Dev. Biol. 289, 127–140. 10.1016/j.ydbio.2005.10.019 16316642

[B26] Hamburger V. Hamilton H. L. (1951). A series of normal stages in the development of the chick embryo. J. Morphol. 88, 49–92. 10.1002/jmor.1050880104 24539719

[B27] He Z. Ma W.-Y. Hashimoto T. Bode A. M. Yang C. S. Dong Z. (2003). Induction of apoptosis by caffeine is mediated by the p53, Bax, and caspase 3 pathways. Cancer Res. 63, 4396–4401. Available online at: https://cancerres.aacrjournals.org/content/63/15/4396.long 12907610

[B28] Henrique D. Adam J. Myat A. Chitnis A. Lewis J. Ish-Horowicz D. (1995). Expression of a Delta homologue in prospective neurons in the chick. Nature 375, 787–790. 10.1038/375787a0 7596411

[B29] Hirsinger E. Duprez D. Jouve C. Malapert P. Cooke J. Pourquié O. (1997). Noggin acts downstream of Wnt and Sonic Hedgehog to antagonize BMP4 in avian somite patterning. Development 124, 4605–4614. 9409677 10.1242/dev.124.22.4605

[B30] Illanes J. Fuenzalida M. Romero S. González P. Lemus D. (1999). Ethanol effect on the chick embryo ossification: a macroscopic and microscopic study. Biol. Res. 32, 77–84. 10883321

[B31] Jang Y.-N. Baik E. J. (2013). JAK-STAT pathway and myogenic differentiation. Jak-Stat 2, e23282. 10.4161/jkst.23282 24058805 PMC3710318

[B32] Kawakami Y. Raya A. Raya R. M. Rodriguez-Esteban C. Izpisua Belmonte J. C. (2005). Retinoic acid signalling links left-right asymmetric patterning and bilaterally symmetric somitogenesis in the zebrafish embryo. Nature 435, 165–171. 10.1038/nature03512 15889082

[B33] Kruger M. Mennerich D. Fees S. Schafer R. Mundlos S. Braun T. (2001). Sonic hedgehog is a survival factor for hypaxial muscles during mouse development. Development 128, 743–752. Available online at: https://dev.biologists.org/content/develop/128/5/743.full.pdf 11171399 10.1242/dev.128.5.743

[B34] Lashein F. E.-D. M. Seleem A. A. Ahmed A. A. (2016). Effect of caffeine and retinoic acid on skeleton of mice embryos. J. Basic Appl. Zool. 75, 36–45. 10.1016/j.jobaz.2016.06.003

[B35] Li X. He R. Qin Y. Tsoi B. Li Y. Ma Z.. (2012). Caffeine interferes embryonic development through over-stimulating serotonergic system in chicken embryo. Food Chem. Toxicol. 50, 1848–1853. 10.1016/j.fct.2012.03.037 22449533

[B36] Linask K. K. Ludwig C. Han M.-D. Liu X. Radice G. L. Knudsen K. A. (1998). N-cadherin/catenin-mediated morphoregulation of somite formation. Dev. Biol. 202, 85–102. 10.1006/dbio.1998.9025 9758705

[B37] Ma Z. Wang G. Cheng X. Chuai M. Kurihara H. Lee K. K. H.. (2014). Excess caffeine exposure impairs eye development during chick embryogenesis. J. Cell. Mol. Med. 18, 1134–1143. 10.1111/jcmm.12260 24636305 PMC4508153

[B38] Ma Z. L. Qin Y. Wang G. Li X. D. He R. R. Chuai M. L.. (2012). Exploring the caffeine-induced teratogenicity on neurodevelopment using early chick embryo. PLoS One 7:e34278. 10.1371/journal.pone.0034278 22470550 PMC3314624

[B39] Ma Z. L. Wang G. Lu W. H. Cheng X. Chuai M. L. Lee K. K. H.. (2016). Investigating the effect of excess caffeine exposure on placental angiogenesis using chicken 'functional' placental blood vessel network. J. Appl. Toxicol. 36, 285–295. 10.1002/jat.3181 26179615

[B40] Mak S. M. Bhaludin B. N. Naaseri S. Di Chiara F. Jordan S. Padley S. (2016). Imaging of congenital chest wall deformities. Br. J. Radiol. 89, 20150595. 10.1259/bjr.20150595 26916279 PMC4985446

[B41] Marcelle C. Stark M. R. Bronner-Fraser M. (1997). Coordinate actions of BMPs, Wnts, Shh and noggin mediate patterning of the dorsal somite. Development 124, 3955–3963. 9374393 10.1242/dev.124.20.3955

[B42] Marret S. Gressens P. Van-Maele-Fabry G. Picard J. Evrard P. (1997). Caffeine-induced disturbances of early neurogenesis in whole mouse embryo cultures. Brain Res. 773, 213–216. 10.1016/S0006-8993(97)00938-4 9409724

[B43] McKinnell I. W. Ishibashi J. Le Grand F. Punch V. G. Addicks G. C. Greenblatt J. F.. (2008). Pax7 activates myogenic genes by recruitment of a histone methyltransferase complex. Nat. Cell Biol. 10, 77–84. 10.1038/ncb1671 18066051 PMC2739814

[B44] Mic F. A. Duester G. (2003). Patterning of forelimb bud myogenic precursor cells requires retinoic acid signaling initiated by Raldh2. Dev. Biol. 264, 191–201. 10.1016/S0012-1606(03)00403-2 14623241

[B45] Miller R. R. Jr Heckel C. D. Koss W. J. Montague S. L. Greenman A. L. (2001). Ethanol-and nicotine-induced membrane changes in embryonic and neonatal chick brains. Comp. Biochem. Physiol. C Toxicol. Pharmacol. 130, 163–178. 10.1016/S1532-0456(01)00227-7 11574286

[B46] Molkentin J. D. Olson E. N. (1996). Combinatorial control of muscle development by basic helix-loop-helix and MADS-box transcription factors. Proc. Natl. Acad. Sci. U. S. A. 93, 9366–9373. 10.1073/pnas.93.18.9366 8790335 PMC38433

[B47] Münsterberg A. Kitajewski J. Bumcrot D. A. McMahon A. P. Lassar A. B. (1995). Combinatorial signaling by Sonic hedgehog and Wnt family members induces myogenic bHLH gene expression in the somite. Genes Dev. 9, 2911–2922. 10.1101/gad.9.23.2911 7498788

[B48] Narod S. A. de Sanjosé S. Victora C. (1991). Coffee during pregnancy: a reproductive hazard? Am. J. Obstet. Gynecol. 164, 1109–1114. 10.1016/0002-9378(91)90597-K 2014836

[B49] Nehlig A. (2018). Interindividual differences in caffeine metabolism and factors driving caffeine consumption. Pharmacol. Rev. 70, 384–411. 10.1124/pr.117.014407 29514871

[B50] Nehlig A. Daval J. L. Debry G. (1992). Caffeine and the central nervous system: mechanisms of action, biochemical, metabolic and psychostimulant effects. Brain Res. 17, 139–170. 10.1016/0165-0173(92)90012-B 1356551

[B51] Pourquié O. (2004). The chick embryo: a leading model in somitogenesis studies. Mech. Dev. 121, 1069–1079. 10.1016/j.mod.2004.05.002 15296972

[B52] Pourquié O. (2018). Somite formation in the chicken embryo. Int. J. Dev. Biol. 62, 57–62. 10.1387/ijdb.180036op 29616740

[B53] Power S. C. Lancman J. Smith S. M. (1999). Retinoic acid is essential for Shh/Hoxd signaling during rat limb outgrowth but not for limb initiation. Dev. Dyn. 216, 469–480. 10.1002/(SICI)1097-0177(199912)216:4/5<469::AID-DVDY15>3.0.CO;2-3 10633866

[B54] Qian J. Chen Q. Ward S. M. Duan E. Zhang Y. (2019). Impacts of caffeine during pregnancy. Trends Endocrinol. Metab. 31, 218–227. 10.1016/j.tem.2019.11.004 31818639 PMC7035149

[B55] Qian J. Zhang Y. Qu Y. Zhang L. Shi J. Zhang X.. (2018). Caffeine consumption during early pregnancy impairs oviductal embryo transport, embryonic development and uterine receptivity in mice. Biol. Reprod. 99, 1266–1275. 10.1093/biolre/ioy155 29982366 PMC6299251

[B56] Reis A. M. S. Raad R. V. de Melo Ocarino N. Serakides R. (2013). *In vitro* effects of caffeine in growth cartilage of rats. Acta Ortoped. Bras. 21, 307. 10.1590/S1413-78522013000600001 24453686 PMC3874990

[B57] Relaix F. Rocancourt D. Mansouri A. Buckingham M. (2005). A Pax3/Pax7-dependent population of skeletal muscle progenitor cells. Nature 435, 948–953. 10.1038/nature03594 15843801

[B58] Resende T. P. Ferreira M. Teillet M.-A. Tavares A. T. Andrade R. P. Palmeirim I. (2010). Sonic hedgehog in temporal control of somite formation. Proc. Natl. Acad. Sci. U. S. A. 107, 12907–12912. 10.1073/pnas.1000979107 20615943 PMC2919945

[B59] Reshef R. Maroto M. Lassar A. B. (1998). Regulation of dorsal somitic cell fates: BMPs and Noggin control the timing and pattern of myogenic regulator expression. Genes Dev. 12, 290–303. 10.1101/gad.12.3.290 9450925 PMC316485

[B60] Roda-Moreno J. Pascual-Morenilla M. Roda-Murillo O. Lopez-Soler M. Arrebola-Nacle F. (2000). Action of ethanol on different skull and brain parameters in the chick embryo. J. Craniofac. Genet. Dev. Biol. 20, 44–48. 10879657

[B61] Sahu S. Kauser H. Ray K. Kishore K. Kumar S. Panjwani U. (2013). Caffeine and modafinil promote adult neuronal cell proliferation during 48 h of total sleep deprivation in rat dentate gyrus. Exp. Neurol. 248, 470–481. 10.1016/j.expneurol.2013.07.021 23920241

[B62] Santos R. M. M. Lima D. R. A. (2016). Coffee health effects from early fetal development through childhood and adolescence, in Translational Toxicology, eds. Hughes C. Waters M. (Cham: Humana Press; Springer), 321–337.

[B63] Sawada K. Sakata-Haga H. Komatsu S. Ohta K. Jeong Y. G. Fukui Y. (2002). A selective loss of small-diameter myelinated optic nerve axons in rats prenatally exposed to ethanol. Congen. Anom. 42, 125–129. 10.1111/j.1741-4520.2002.tb00861.x 12196709

[B64] Shum A. S. Poon L. L. Tang W. W. Koide T. Chan B. W. Leung Y.-C. G.. (1999). Retinoic acid induces down-regulation of Wnt-3a, apoptosis and diversion of tail bud cells to a neural fate in the mouse embryo. Mech. Dev. 84, 17–30. 10.1016/S0925-4773(99)00059-3 10473117

[B65] Sirbu I. O. Duester G. (2006). Retinoic-acid signalling in node ectoderm and posterior neural plate directs left-right patterning of somitic mesoderm. Nat. Cell Biol. 8, 271–277. 10.1038/ncb1374 16489341 PMC2805411

[B66] Song J. Wang C. Long D. Li Z. You L. Brand-Saberi B.. (2020). Dysbacteriosis-induced LPS elevation disturbs the development of muscle progenitor cells by interfering with retinoic acid signaling. FASEB J. 34, 6837–6853. 10.1096/fj.201902965R 32223025

[B67] Teillet M. Watanabe Y. Jeffs P. Duprez D. Lapointe F. Le Douarin N. (1998). Sonic hedgehog is required for survival of both myogenic and chondrogenic somitic lineages. Development 125, 2019–2030. 9570767 10.1242/dev.125.11.2019

[B68] Umemoto T. Furutani Y. Murakami M. Matsui T. Funaba M. (2011). Endogenous Bmp4 in myoblasts is required for myotube formation in C2C12 cells. Biochim. Biophys. Acta 1810, 1127–1135. 10.1016/j.bbagen.2011.09.008 21964441

[B69] Vermot J. Gallego Llamas J. Fraulob V. Niederreither K. Chambon P. Dolle P. (2005). Retinoic acid controls the bilateral symmetry of somite formation in the mouse embryo. Science 308, 563–566. 10.1126/science.1108363 15731404

[B70] Wang G. Jiang L. M. Tan B. Y. Li P. Z. Zhang P. L. Zhang Y.. (2019). Cell survival controlled by lens-derived Sema3A-Nrp1 is vital on caffeine-suppressed corneal innervation during chick organogenesis. J. Cell. Physiol. 234, 9826–9838. 10.1002/jcp.27671 30362583

[B71] Wang G. Li Y. Wang X. Y. Chuai M. Yeuk-Hon Chan J. Lei J.. (2015). Misexpression of BRE gene in the developing chick neural tube affects neurulation and somitogenesis. Mol Biol Cell 26, 978–992. 10.1091/mbc.E14-06-1144 25568339 PMC4342032

[B72] Warkman A. S. Whitman S. A. Miller M. K. Garriock R. J. Schwach C. M. Gregorio C. C.. (2012). Developmental expression and cardiac transcriptional regulation of Myh7b, a third myosin heavy chain in the vertebrate heart. Cytoskeleton 69, 324–335. 10.1002/cm.21029 22422726 PMC4734749

[B73] Wilkinson J. M. Pollard I. (1993). Accumulation of theophylline, theobromine and paraxanthine in the fetal rat brain following a single oral dose of caffeine. Dev. Brain Res. 75, 193–199. 10.1016/0165-3806(93)90023-4 8261611

[B74] Wink C. Rossowska M. Nakamoto T. (1996). Effects of caffeine on bone cells and bone development in fast-growing rats. Anat. Rec. 246, 30–38. 10.1002/(SICI)1097-0185(199609)246:1<30::AID-AR4>3.0.CO;2-J 8876821

[B75] Yadegari M. Khazaei M. Anvari M. Eskandari M. (2016). Prenatal caffeine exposure impairs pregnancy in rats. Int. J. Fertil. Steril. 9, 558–562. 10.22074/ijfs.2015.4616 26985345 PMC4793178

[B76] Yu T. Campbell S. C. Stockmann C. Tak C. Schoen K. Clark E. A.. (2016). Pregnancy-induced changes in the pharmacokinetics of caffeine and its metabolites. J. Clin. Pharmacol. 56, 590–596. 10.1002/jcph.632 26358647 PMC5564294

[B77] Zulli A. Smith R. M. Kubatka P. Novak J. Uehara Y. Loftus H.. (2016). Caffeine and cardiovascular diseases: critical review of current research. Eur. J. Nutr. 55, 1331–1343. 10.1007/s00394-016-1179-z 26932503

