# Supplementary material for: Retinoic Acid Signaling Plays a Crucial Role in Excessive Caffeine Intake-Disturbed Apoptosis and Differentiation of Myogenic Progenitors
Source: Front Cell Dev Biol. 2021 Mar 9;9:586767. doi: 10.3389/fcell.2021.586767 (PMC8006404; doi:10.3389/fcell.2021.586767)
Supplement: Supplementary file 1 [file Data_Sheet_1.doc]

**Supplemental Material**

**Table S1. The primers used in this study.**

| **Primers for quantitative PCR** | | |
| --- | --- | --- |
|  | **Forward primer (5’-3’)** | **Reverse Primer (5’-3’)** |
| MYH7B(Chicken) | CCAGGAGCTCTTGAGGGAAC | AGTCCGCCATCACATCGGAG |
| MEF2A(Chicken) | GAAACGGAGCCTTTTGGGCTT | CTTTCCCTTCCTTTGCAATCCAG |
| myogenin(Chicken) | CACCAACCCCGCAGATCAC | TTCCAGCATCACCATCCCAC |
| Myod1(Chicken) | GGGAGTCGGGAACCCTCC | AGGTCTCACCCACCTCTGG |
| Pax7(Chicken) | CGGACATCTACACCAGGGAGGAG | ATCTTGCTCGTCGGTTGCTGAAC |
| Myf5(Chicken) | CCAGGAGCTCTTGAGGGAAC | AGTCCGCCATCACATCGGAG |
| BMP4(Chicken) | GATCCGCTTCGTCTTCAACCTCAG | CTCGGCTCCTCCACCTGCTC |
| RALDH2(Chicken) | ATTCCTGCAAGCCTTCTACG | ATTCCTGCAAGCCTTCTACG |
| GAPDH(Chicken) | GGGCACGCCATCACTATCTT | TCACAAACATGGGGGCATCA |
| Shh(Chicken) | CCTGGCCAACGTGTCTATGT | CAACACCCGGTTGATGAGGA |
| Wnt3a(Chicken)  Caspase3(Chicken)  Cyclin D1(Chicken)  Caspase3(Mouse)  Cyclin D1(Mouse)  RALDH2(Mouse)  BMP4(Mouse)  Shh(Mouse)  GAPDH(Mouse) | TCATCCCGCCTCGGAAGAAA  TGGTGGAGGTGGAGGAGC  CACTTGGATGCTGGAGGTCTG  GAAACTCTTCATCATTCAGGCC  CGTATCTTACTTCAAGTGCGTG  ATGTTCACCTGGAAAATTGCTC  CGAATGCTGATGGTCGTTTTAT  GAAGGTCTTCTACGTGATCGAG  TCAAATGGGCAGATGCAGGT | AGAGCCTGACTCAACCCACA  GCGTGGTCCATCTTTTATGTCT  GCACAGTTTTTCTGCGGTCA  GCGAGTGAGAATGTGCATAAAT  ATGGTCTCCTTCATCTTAGAGG  CAATCTTGTCTATGCCGATGTG  GATCCCTCATGTAATCCGGAAT  TTCAGCCACCACGTACAC  AGCTGAGGGAGCTGAGATGA |

| **Primers for *in situ* hybridization** | | |
| --- | --- | --- |
|  | **Forward primer (5’-3’)** | **Reverse Primer (5’-3’)** |
| RALDH2 | ATTCCTGCAAGCCTTCTACG | TTGCTCCTTCAGTAATGCCG |
| Myf5 | GAAGGCAGCCACTATGAGGG | ATAGCGCCTGGTAGGTCCG |

**
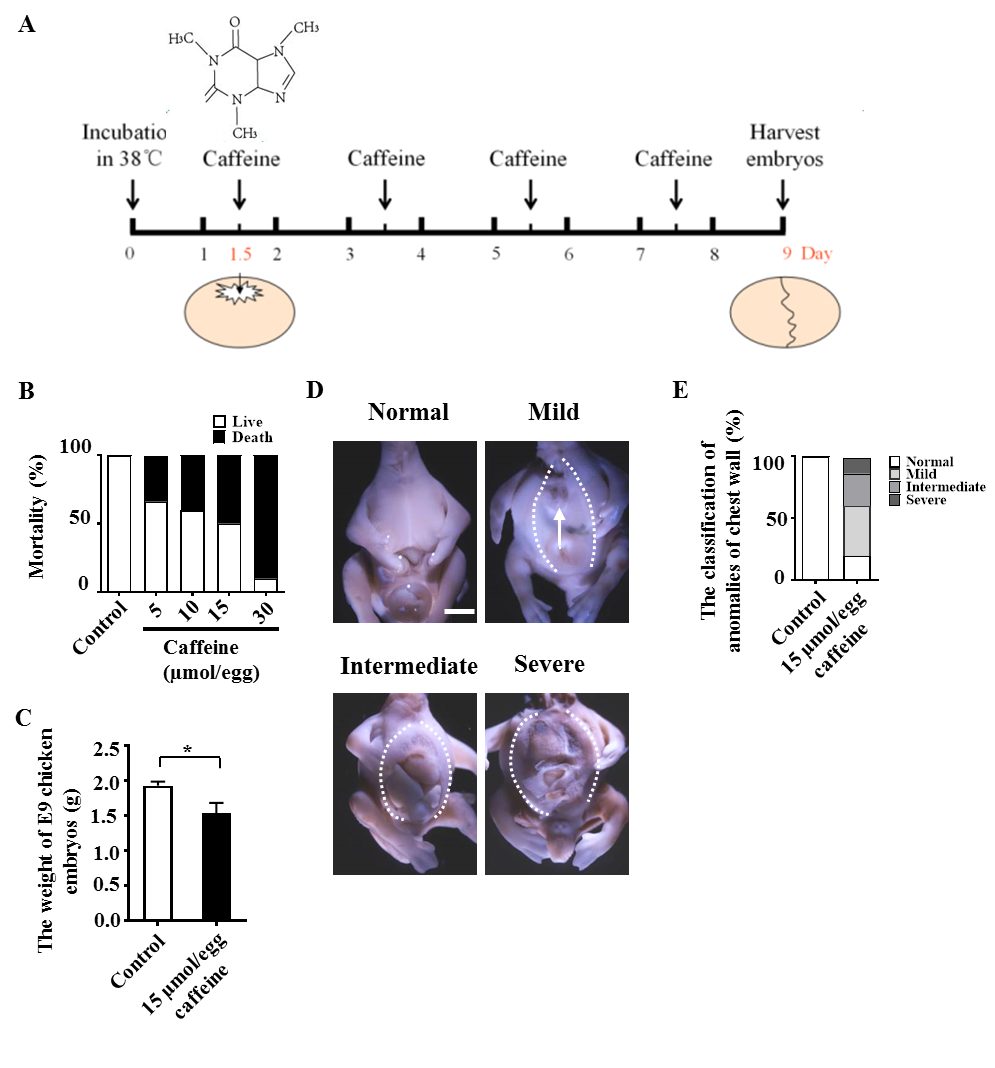
Figure S1. Assessment of the development of chicken embryos treated with caffeine. (A)** The sketch illustrates how E1.5 (HH10) chicken embryos were treated with either PBS or caffeine and then harvested on Day 9. **(B-C)** The bar charts showing the comparisons of E9 chicken embryo mortality **(B)**, and embryo weight **(C)** between control and caffeine-treated groups. **(D)** The representative E9 chicken embryos from 15 μmol/egg caffeine-treated group, with normal chest wall, mild, intermediate or severe unclosed chest wall. **(E)** The bar chart showing the comparisons of E9 chicken embryos in classification of abnormal chest wall between control and 15 μmol/egg caffeine-treated groups. Scale bars=200 μm in **(D)**.

**
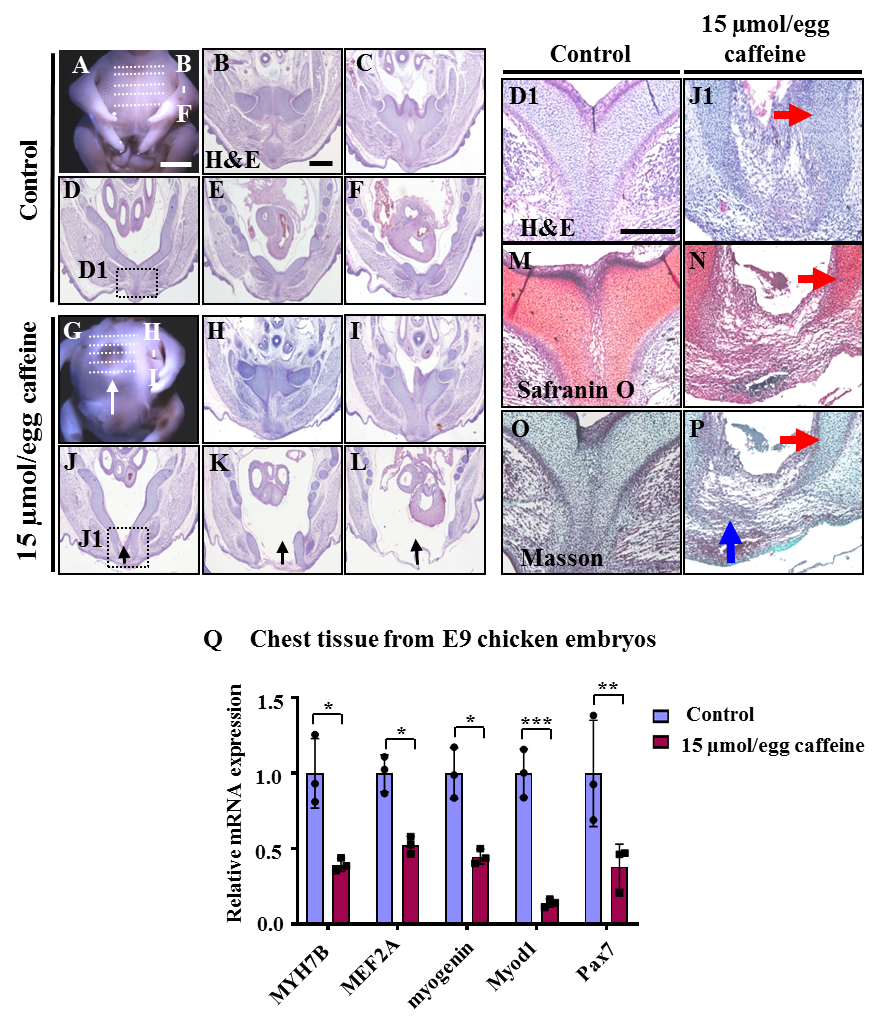
**

**Figure S2. Assessment of myogenesis in E9 chicken embryos following 15 μmol/egg caffeine treatment. (A, G)** Whole images of the chicken embryos from the control and 15 μmol/egg caffeine-treated groups. The white arrow represent the unclosed chest wall in **(G)**. **(B-F)** and **(H-L)** H&E staining was performed on transverse sections of the chest wall indicated by dotted lines in **(A)** and **(G)**. The black arrows represent the areas of unclosed chest wall in **(J-L)**, corresponding to the areas represented by the white arrow in **(G)**. **(D1, J1, M, N, O, P)** High magnification image from the sites indicated by **(D)** and **(J)**, respectively, which were stained by H&E **(D1, J1)**, Safranin O **(M, N)** and Masson **(O, P)**. The red arrows represent the areas of cartilage in **(J1, N, P)**, while the blue arrow represents the areas of muscle tissue in **(P)**. **(Q)** Quantitative RT-PCR data showing the mRNA expression of MYH7B, MEF2A, myogenin, Myod1 and Pax7. Scale bars=200 μm in A and G; 100 μm in **(B-F)** and **(H-L)**; 100 μm in **(D1**, **J1**and **M-P)**.

**
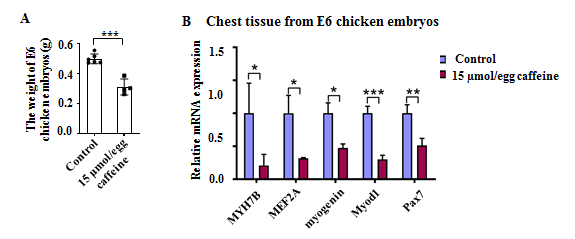
**

**Figure S3. Assessment of myogenesis in E6 chicken embryos following 15 μmol/egg caffeine treatment.** (A) The bar chart showing the comparisons of chicken embryo weight between the control and caffeine-treated groups. (B) Quantitative RT-PCR data showing the mRNA expression of MYH7B, MEF2A, myogenin, Myod1 and Pax7.

**
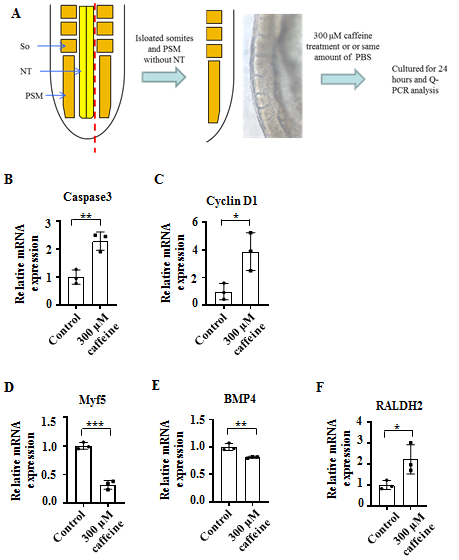
**

**Figure S4. Quantitative RT-PCR data on determining the somitogenesis-related genes following caffeine treatment.** **(A)** The sketch illustrates the development of isolated somites and PSM with or without caffeine treatment. **(B)** Quantitative RT-PCR data showing the mRNA expression of Caspase3, Cyclin D1, Myf5, BMP4 and RALDH2. NT, neural tube; So, somite; PSM, presomitic mesoderm.

**
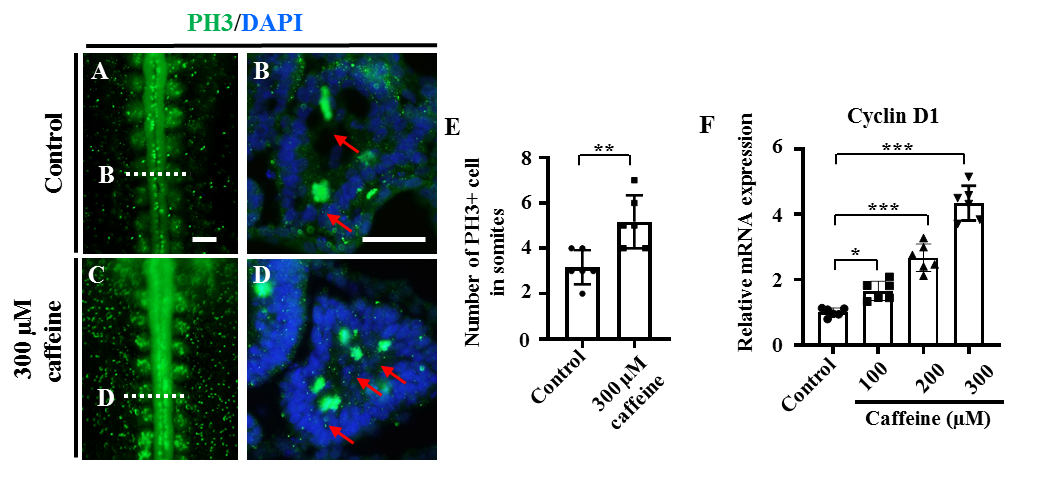
Figure S5. Determination of cell proliferation in the developing somites of HH10 embryos and C2C12 cells following caffeine treatment. (A-D)** Immunofluorescent staining of PH3 in the chicken embryos from the control **(A)** and caffeine-treated **(C)** groups, and the corresponding transverse sections for PH3 and merge with DAPI staining **(B, D)**. **B/D** is from the 5^th^ pair of somites. **(E)** The bar chart showing the proportion of PH3 positive cell numbers in somite from the control and caffeine-treated groups. **(F)** Quantitative RT-PCR data showing the mRNA expression of Cyclin D1 in C2C12 cells after caffeine treatment. Scale bars=100 μm in **(A,C)**; 50 μm in **(B, D)**.

**
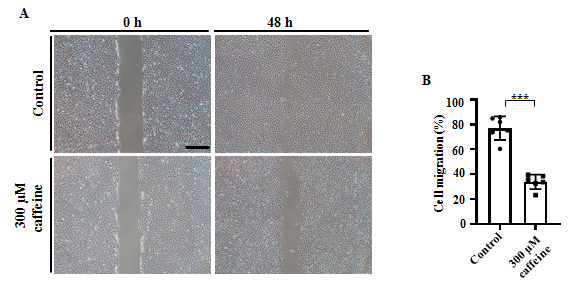
**

**Figure S6. Assessment of the cell migration ability of C2C12 myoblast cells following 48 h treatment of caffeine. (A)** Representative images of transwell migration assay from the control and caffeine-treated groups were taken after incubating C2C12 with caffeine for 0 h and 48 hours. **(B)** The bar charts showing the comparisons of cell migration ratios between the control and caffeine-treated groups. Scale bars= 300 μm in **(A)**.

**
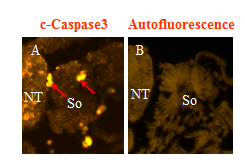
**

**Figure S7. The negative control of c-Caspase3 immunofluorescence. (A)** Immunofluorescent staining of c-Caspase3 in the chicken embryos. **(B)** The negative control of c-Caspase3 immunofluorescence. NT, neural tube; So, somite.

**More details on statistics of all the figures included**

**Figure S1:**

**B:** **Statistical analysis of mortality rate (%) of E9 chicken embryos:**

Control: live: 100%, death: 0%, 60 embryos counted;

5 µM: live: 67%, death: 33%, 60 embryos counted;

10 µM: live: 60 %, death: 40%, 60 embryos counted;

15 µM: live: 50%, death: 50 %, 60 embryos counted;

30 µM: live: 10%, death: 90%, 60 embryos counted.

**C: Statistical analysis of the weight (g) of E9 chicken embryos by independent samples *t* test:**

Control: 1.9242 ± 0.31226, 28 embryos analyzed; Caffeine: 1.5364 ± 0.60328, 17 embryos analyzed. There is significant difference between the groups (p＜0.05, *).

**E: Statistical analysis of the anomaly ratio (%) of chest wall :**

Control: normal: 100%, mild: 0%, intermediate: 0%, severe: 0%, 30 embryos analyzed;

Caffeine: normal: 20%, mild: 40%, intermediate: 26%, severe: 13%, 30 embryos analyzed

.

**Figure S2:**

**Q: Statistical analysis of the** **relative mRNA expression of chest tissue from E9 chicken embryo by independent samples *t* test.** The RT-PCR result was representative of at least three independent experiments:

MYH7B: Control: 1.0 ± 0.230245; Caffeine: 0.393034 ± 0.042935. There is significant difference between the groups (p＜0.05, *).

MEF2A: Control: 1.0 ± 0.123021, Caffeine: 0.524485 ± 0.05736. There is significant difference between the groups (p＜0.01, **).

myogenin：Control: 1.0 ± 0.168876; Caffeine: 0.44757 ± 0.049486. There is significant difference between the groups ( p＜0.01, **).

Myod1: Control: 1.0 ± 0.159814; Caffeine: 0.140795 ± 0.026718. There is significant difference between the groups ( p＜0.001, ***).

Pax7: Control: 1.0 ± 0.352073, Caffeine: 0.380319 ± 0.150462. There is significant difference between the groups ( p＜0.05, *).

**Figure S3:**

**A: Statistical analysis of weight of E6 chicken embryo (g) by independent samples *t* test:**

Control: 0.499000 ± 0.0320974, 6 embryos analyzed; Caffeine: 0.311300 ± 0.0533819, 4 embryos analyzed. There is significant difference between the groups (p＜0.001, ***).

**B: Statistical analysis of the** **relative mRNA expression of chest tissue from E6 chicken embryo by independent samples *t* test.** The RT-PCR result was representative of at least three independent experiments:

MYH7B: Control: 1.0 ± 0.456363; Caffeine: 0.207328 ± 0.168904. There is significant difference between the groups (p＜0.05,*).

MEF2A: Control: 1.0 ± 0.270431; Caffeine: 0.317569 ± 0.010237. There is significant difference between the groups (p＜0.05, *).

myogenin: Control: 1.0 ± 0.157457; Caffeine: 0.472968 ± 0.060514. There is significant difference between the groups (p＜0.05, *).

Myod1: Control: 1.0 ± 0.105052; Caffeine: 0.301665 ± 0.060797. There is significant difference between the groups (p＜0.001, ***).

Pax7: Control: 1.0 ± 0.129500; Caffeine: 0.512500 ± 0.106640. There is significant difference between the groups (p＜0.01, **).

**Figure S4:**

**B: Statistical analysis of somite from HH10 chicken embryo by independent samples *t* test.** The RT-PCR result was representative of at least three independent experiments:

Caspase3: Control: 1.0000 ± 0.32250; Caffeine: 3.4722 ± 0.30582. There is significant difference between the groups (p＜0.001, ***).

**C: Statistical analysis of somite from HH10 chicken embryo by independent samples *t* test.** The RT-PCR result was representative of at least three independent experiments:

Cyclin D1: Control: 1.0000 ± 0.12975; Caffeine: 3.5704 ± 0.86986; There is significant difference between the groups (p＜0.01, **).

**D: Statistical analysis of somite from HH10 chicken embryo by independent samples *t* test.** The RT-PCR result was representative of at least three independent experiments:

Myf5: Control: 1.0000 ± 0.09656; Caffeine: 0.6577 ± 0.15505; There is significant difference between the groups (p＜0.05, *).

**E: Statistical analysis of somite from HH10 chicken embryo by independent samples *t* test.** The RT-PCR result was representative of at least three independent experiments:

BMP4: Control: 1.0000 ± 0.17088; Caffeine: 0.3322 ± 0.08528; There is significant difference between the groups (p＜0.01, **).

**F: Statistical analysis of somite from HH10 chicken embryo by independent samples *t* test.** The RT-PCR result was representative of at least three independent experiments:

RALDH2: Control: 1.0000 ± 0.36104; Caffeine: 2.8540 ± 0.85093; There is significant difference between the groups (p＜0.05, *).

**Figure S5:**

**E: Statistical analysis of number of PH3+ cells in somite by independent samples *t* test:**

Control: 3.5000 ± 1.04881, a total of three embryos (six sections) were analyzed; Caffeine: 5.1667 ± 1.16905, a total of three embryos (six sections) were analyzed. There is significant difference between the groups (p＜0.05, *).

**F:** **Statistical analysis of relative mRNA expression of C2C12 cell by Turkey’s multiple comparisons test.** The RT-PCR result was representative of at least three independent experiments:

CyclinD1: Control: 1.0000 ± 0.13057; 100µM Caffeine: 1.6571 ± 0.29281; 200µM Caffeine 2.6725 ± 0.42062; 300µM Caffeine: 4.3554 ± 0.53082. There is significant difference between 100µM Caffeine group and Control group (p＜0.05, *). There is significant difference between 200µM Caffeine group and Control group (p＜0.001, ***). There is significant difference between 300µM Caffeine group and Control group (p＜0.001, ***).

**Figure S6:**

**B: Statistical analysis of cell migration rate by independent samples *t* test:**

Control: 0.7697 ± 0.09535; Caffeine: 0.3388 ± 0.05843; There is significant difference between the groups (p＜0.001, ***)

**Figure 1:**

**C: Statistical analysis of mortality rate (%)：**

Control: 12h: 0%; 18h: 0%; 38h: 0%, 9 embryos counted;

Caffeine: 12h: 0%; 18h: 15%; 38h: 37%, 16 embryos counted;

**D: Statistical analysis of the** **length of embryos (mm) by independent samples *t* test.**

12h: Control: 2.076111 ± 0.0448426, 9 embryos analyzed; Caffeine: 12h: 2.050875 ±0.038593, 16 embryos analyzed. There is no significant difference between the groups (p＞0.05, NS).

18h: Control: 2.577500 ± 0.2094039, 8 embryos analyzed; Caffeine: 2.2669 ± 0.10094, 14 embryos analyzed. There is significant difference between the groups (p＜0.01, **).

34h: Control: 4.484286 ± 0.1820126, 7 embryos analyzed; Caffeine: 3.870769 ± 0.2092232, 13 embryos analyzed. There is significant difference between the groups ( p＜0.001, ***).

**E: Statistical analysis of the pairs of somites at 34 h-incubated chicken embryos by independent samples *t* test**.

Control: 9.571429 ± 1.2392394, 7 embryos analyzed; Caffeine: 7.833333 ± 1.3291601, 6 embryos analyzed. There is significant difference between the groups (p＜0.05, *).

**L: Statistical analysis of the** **relative mRNA expression by independent samples *t* test.** The RT-PCR result was representative of at least three independent experiments

Pax7: Control: 1.0 ± 0.05492; Caffeine: 0.5981 ± 0.06049. There is significant difference between the groups (p＜0.01, **).

**Figure 2:**

**E: Statistical analysis of the** **number of c-Caspase3+ cells in somite by independent samples *t* test:**

Control: 2.250000 ± 2.5495098, a total of three embryos (eight sections) were analyzed; Caffeine: 9.750000 ± 2.9640706, a total of three embryos (eight sections) were analyzed. There is significant difference between the groups (p＜0.001, ***).

**F: Statistical analysis of relative protein level of c-Caspase3 by independent samples *t* test:**

Control: 1.0702 ± 0.12558; Caffeine: 2.6176 ± 0.42646. There is significant difference between the groups (p＜0.01, **).

**G: Statistical analysis of the** **relative mRNA expression of C2C12 cell by Turkey’s multiple comparisons test.** The RT-PCR result was representative of at least three independent experiments:

Caspase3: Control: 1.0000 ± 0.23535; 100µM Caffeine: 1.8760 ± 0.38317; 200µM Caffeine 2.6243 ± 0.72528; 300µM Caffeine: 2.7111 ± 0.64484. There is significant difference between 100µM Caffeine group and Control group (p＜0.05, *). There is significant difference between 200µM Caffeine group and Control group (p＜0.001, ***). There is significant difference between 300µM Caffeine group and Control group (p＜0.001, ***)

**Figure 3:**

**E:** **Statistical analysis of the** **relative mRNA expression by independent samples *t* test.**The RT-PCR result was representative of at least three independent experiments:

Myf5: Control: 1.0 ± 0.05398; Caffeine: 0.0518 ± 0.00960. There is significant difference between the groups (p＜0.001, ***).

**L: Statistical analysis of the** **relative mRNA expression by independent samples *t* test.**The RT-PCR result was representative of at least three independent experiments:

BMP4: Control: 1.0 ± 0.05496; Caffeine: 0.1793 ± 0.04234. There is significant difference between the groups (p＜0.001, ***).

**M: Statistical analysis of the** **relative mRNA expression by independent samples *t* test.**The RT-PCR result was representative of at least three independent experiments

Wnt3a: Control: 1.0000 ± 0.07683; Caffeine: 0.6979 ± 0.12614. There is significant difference between the groups (p＜0.05, *)

**N: Statistical analysis of the** **relative mRNA expression by independent samples *t* test.** The RT-PCR result was representative of at least three independent experiments

Shh: Control: 1.0000 ± 0.06727; Caffeine: 1.7616 ± 0.14036. There is significant difference between the groups ( p＜0.01, **)

**Figure 4:**

**A: Statistical analysis of the** **concentration of RA(ng/mg) by independent samples *t* test:**

Control: 0.264231267 ± 0.0280459442; Caffeine: 0.344258567 ± 0.0151073625. 15 embryos were mixied for 1 simple, N>3 simples in each group. There is significant difference between the groups (p＜0.05, *).

**B:**  **Statistical analysis of the** **relative mRNA expression by independent samples *t* test.** The RT-PCR result was representative of at least three independent experiments:

RALDH2: Control: 1.0 ± 0.13178; Caffeine: 1.4541 ± 0.21102. There is significant difference between the groups (p＜0.05, *).

**B1：Statistical analysis of the** **relative mRNA expression of C2C12 cell by Turkey’s multiple comparisons test.** The RT-PCR result was representative of at least three independent experiments:

RALDH2: Control: 1.0000 ± 0.19400; 100µM Caffeine: 1.9975 ± 0.30960; 200µM Caffeine 2.3960 ± 0.17861; 300µM Caffeine: 2.8336 ± 0.61432. There is significant difference between 100µM Caffeine group and Control group (p＜0.01, **). There is significant difference between 200µM Caffeine group and Control group (p＜0.001, ***). There is significant difference between 300µM Caffeine group and Control group (p＜0.001, ***).

**H：Statistical analysis of the immunofluorescence staining area of somite from** **E2.5 chicken embryos** **by Turkey’s multiple comparisons test.**

MF20: Control: 28008.6508 ± 2854.11255, 6 embryos analyzed; Caffeine: 14528.4222 ± 1542.98298, 6 embryos analyzed; Caffeine + AGN: 9405.4907 ± 937.45110, 6 embryos analyzed. There is significant difference between Caffeine group and Control group (p＜0.001, ***). There is significant difference between Caffeine group and Caffeine + AGN group (p＜0.001, ***).

**Figure 5:**

**B: Statistical analysis of Pairs of somites at 38h chicken embryo by independent samples *t* test:**

Control: 11.8148 ± 0.87868, 27 embryos analyzed; Caffeine: 8.5000 ± 1.76608, 22 embryos analyzed; Caffeine + AGN: 9.8000 ± 1.25831, 25 embryos analyzed. There is significant difference between Caffeine group and Control group (p＜0.01, **). There is significant difference between Caffeine group and Caffeine + AGN group (p＜0.05 , *).

**Figure 6:**

**A: Statistical analysis of the** **relative mRNA expression of C2C12 cell by Turkey’s multiple comparisons test.** The RT-PCR result was representative of at least three independent experiments:

Caspase3: Control: 1.0000 ± 0.09651; 25µM Caffeine: 5.5905 ± 0.31243; 25µM Caffeine + 10^-5^ M AGN: 1.4354 ± 0.21453; 50µM Caffeine: 7.7227 ± 1.33177; 50µM Caffeine + 10^-5^ M AGN: 2.5458 ± 0.37264. There is significant difference between 25µM Caffeine group and Control group (p＜0.001, *** ). There is significant difference between 25µM Caffeine + 10^-5^ M AGN group and 25µM Caffeine group (p＜0.001, ***). There is significant difference between 50µM Caffeine group and Control group (p＜0.001, *** ). There is significant difference between 50µM Caffeine + 10^-5^ M AGN group and 50µM Caffeine group (p＜0.001, ***).

**B: Statistical analysis of the** **relative mRNA expression of C2C12 cell by Turkey’s multiple comparisons test.** The RT-PCR result was representative of at least three independent experiments:

CyclinD1: Control: 1.0000 ± 0.36540; 25µM Caffeine: 5.8654 ± 0.72837; 25µM Caffeine + 10^-5^ M AGN: 2.5681 ± 0.15468; 50µM Caffeine: 10.8297 ± 1.71153; 50µM Caffeine + 10^-5^ M AGN: 3.7820 ± 0.24034. There is significant difference between 25µM Caffeine group and Control group (p＜0.001, *** ). There is significant difference between 25µM Caffeine + 10^-5^ M AGN group and 25µM Caffeine group (p＜0.01, **). There is significant difference between 50µM Caffeine group and Control group (p＜0.001, *** ). There is significant difference between 50µM Caffeine + 10^-5^ M AGN group and 50µM Caffeine group (p＜0.001, ***).

**C: Statistical analysis of the** **relative mRNA expression of C2C12 cell by Turkey’s multiple comparisons test.** The RT-PCR result was representative of at least three independent experiments:

Myf5: Control: 1.0000 ± 0.05369; 25µM Caffeine: 0.1332 ± 0.02006; 25µM Caffeine + 10^-5^ M AGN: 0.7685 ± 0.16540; 50µM Caffeine: 0.0952 ± 0.00923; 50µM Caffeine + 10^-5^ M AGN: 0.4337 ± 0.14522. There is significant difference between 25µM Caffeine group and Control group (p＜0.001, *** ). There is significant difference between 25µM Caffeine + 10^-5^ M AGN group and 25µM Caffeine group (p＜0.001, ***). There is significant difference between 50µM Caffeine group and Control group (p＜0.001, *** ). There is significant difference between 50µM Caffeine + 10^-5^ M AGN group and 50µM Caffeine group (p＜0.05, *).

**D: Statistical analysis of the** **relative mRNA expression of C2C12 cell by Turkey’s multiple comparisons test.** The RT-PCR result was representative of at least three independent experiments:

BMP4: Control: 1.0000 ± 0.13432; 25µM Caffeine: 0.1278 ± 0.01239; 25µM Caffeine + 10^-5^ M AGN: 0.8270 ± 0.05128; 50µM Caffeine: 0.0955 ± 0.01203; 50µM Caffeine + 10^-5^ M AGN: 0.4344 ± 0.01041. There is significant difference between 25µM Caffeine group and Control group (p＜0.001, *** ). There is significant difference between 25µM Caffeine + 10^-5^ M AGN group and 25µM Caffeine group (p＜0.001, ***). There is significant difference between 50µM Caffeine group and Control group (p＜0.001, *** ). There is significant difference between 50µM Caffeine + 10^-5^ M AGN group and 50µM Caffeine group (p＜0.01, **).

**E: Statistical analysis of the** **relative mRNA expression of C2C12 cell by Turkey’s multiple comparisons test.** The RT-PCR result was representative of at least three independent experiments:

RALDH2: Control: 1.0000 ± 0.40329; 25µM Caffeine: 2.5789 ± 0.45990; 50µM Caffeine: 4.1102 ± 0.48665; There is significant difference between 25µM Caffeine group and Control group (p＜0.05, * ). There is significant difference between 50µM Caffeine group and Control group (p＜0.001, *** ).
